# Association of RASSF1A, DCR2, and CASP8 Methylation with Survival in Neuroblastoma: A Pooled Analysis Using Reconstructed Individual Patient Data

**DOI:** 10.1155/2020/7390473

**Published:** 2020-12-15

**Authors:** Waleed M. Hassan, Mohamed S. Bakry, Timo Siepmann, Ben Illigens

**Affiliations:** ^1^Dresden International University, Division of Health Care Sciences, Center for Clinical Research and Management Education, Dresden 01067, Germany; ^2^Research Department, Children's Cancer Hospital Egypt 57357, Cairo 11588, Egypt; ^3^Department of Neurology, University Hospital Carl Gustav Carus, Technische Universität Dresden, Dresden 01307, Germany; ^4^Department of Neurology, Beth Israel Deaconess Medical Center, Harvard Medical School, Boston, MA 02215, USA

## Abstract

Neuroblastoma (NB) is a heterogeneous tumor affecting children. It shows a wide spectrum of clinical outcomes; therefore, development of risk stratification is critical to provide optimum treatment. Since epigenetic alterations such as DNA methylation have emerged as an important feature of both development and progression in NB, in this study, we aimed to quantify the effect of methylation of three distinct genes (RASSF1A, DCR2, and CASP8) on overall survival in NB patients. We performed a systematic review using PubMed, Embase, and Cochrane libraries. Individual patient data was retrieved from extracted Kaplan–Meier curves. Data from studies was then merged, and analysis was done on the full data set. Seven studies met the inclusion criteria. Methylation of the three genes had worse overall survival than the unmethylated arms. Five-year survival for the methylated arm of RASSF1A, DCR2, and CASP8 was 63.19% (95% CI 56.55-70.60), 57.78% (95% CI 47.63-70.08), and 56.39% (95% CI 49.53-64.19), respectively, while for the unmethylated arm, it was 93.10% (95% CI 87.40–99.1), 84.84% (95% CI 80.04-89.92), and 83.68% (95% CI 80.28-87.22), respectively. In conclusion, our results indicate that in NB patients, RASSF1A, DCR2, and CASP8 methylation is associated with poor prognosis. Large prospective studies will be necessary to confirm definitive correlation between methylation of these genes and survival taking into account all other known risk factors. (PROSPERO registration number CRD42017082264).

## 1. Introduction

Neuroblastoma (NB) is a heterogeneous tumor affecting the normal development of the adrenal medulla and paravertebral sympathetic ganglia in early childhood [1, 2]. The age-standardized rate of NB is 10.4 per million person-years in children aged 0-14 based on data from the largest cancer registry study including 153 registries from 62 countries [3]. The peak incidence age of NB is in the first year, with very few cases reported after the age of 10 years [4–6]. NB is considered a major cause of pediatric cancer mortality accounting for around 15% of all deaths [7].

The overall impact of known environmental exposures on the etiology of NB is very low, and the consistent incidence rates of neuroblastoma in children across different regions in the world support the hypothesis of a major role of genetic factors [3, 6]. NB shows a wide spectrum of clinical outcomes, since most of the tumors can regress spontaneously while some may be aggressive [8, 9]. This is mainly caused by the difference in biological and molecular features. Hence, NB risk stratification to predict clinical outcomes is continuously evolving [10]. Many earlier studies were aimed at assessing the prognostic power of different genetic mutations in NB [2].

The most common factors associated with the outcome and currently involved in determining the risk of patients are age at diagnosis, stage at diagnosis, MYCN amplification, histopathological classification, and DNA ploidy status [10–14]. The use of these variables in risk stratification has led to defining two subgroups. The first is the low-risk group in which most of the tumors regress spontaneously or with minimal treatment. The second is the high-risk group in which most of the tumors have unfavorable outcomes and will require intensive therapy. Not all of the patients in each group show the same pattern of the disease. Moreover, there are some less well-defined groups between both groups. This may cause some of the patients to be overtreated or undertreated [15–18]. Therefore, additional prognostic factors may be needed in order to refine the risk stratification of NB [15, 19].

Epigenetic alterations such as DNA methylation have emerged as an important feature of both development and progression in many cancers. Methylation of promoter CpG islands is known to inhibit transcriptional initiation and cause permanent silencing of downstream genes [20]. The most common known epigenetic alterations associated with the outcome in NB are methylation of RASSF1A, DCR2, and CASP8 genes [21–24].

In this study, we quantitatively reviewed the effect of DNA methylation of three distinct genes (RASSF1A, DCR2, and CASP8) and their relation with the outcome using individual reconstructed patient data.

## 2. Methods

### 2.1. Search Criteria

We searched available studies in PubMed, Embase, and Cochrane libraries through 1 October 2020 using the following search statement: (neuroblastoma OR nb OR nbl) AND (RASSF1A OR DCR2 OR CASP8 OR “decoy receptor 2” OR “caspase 8”). Studies were included if they met the following inclusion criteria: (1) studies were conducted on neuroblastoma patients, (2) overall survival curves were reported for at least one of the studied genes according to the methylation status, (3) results for each gene were reported separately, and (4) methylation status was measured from tumor samples. Two independent reviewers conducted the search. Discrepancies were resolved based on discussion.

### 2.2. Data Extraction

For each included paper, Kaplan–Meier (KM) curves for the included genes were extracted. The curves were digitized using the software developed by AmsterCHEM ScanIt [25]. ScanIt is a free digitizer program that is used to extract data from graphs by automatic tracing of lines and converting them to numerical coordinates. Individual patient data (IPD) was then reconstructed using an iterative algorithm developed by Guyot et al. [26]. The algorithm utilizes the coordinates extracted by the digitizer software and the total number at risk in each arm to solve the KM equations originally used to develop the graph. The algorithm assumes constant censoring (noninformative). Numbers at risk at different points will be used when reported to increase the accuracy of the algorithm.

### 2.3. Statistical Analysis

The code developed by Guyot et al. was used to retrieve IPD of each KM. Data retrieved from each KM was redrawn and checked visually if the original and the redrawn KM are similar. All the retrieved IPD data were then merged in one file. The aggregated data was then used to construct a KM for each gene. A chi-square log-rank test was then performed to test the statistical significance of the survival difference between both arms. 5-year survival probability was then reported. A multivariable Cox proportional hazard model was used to determine the significance of methylation status adjusting to the paper from which the data has been extracted. The adjusted hazard ratio (HR) was reported with associated 95% confidence intervals (CI). All the statistical analyses were done using R (version 3.4.3) [27].

This review protocol was registered in the Prospero database (CRD42017082264).

## 3. Results

**Figure 1:**
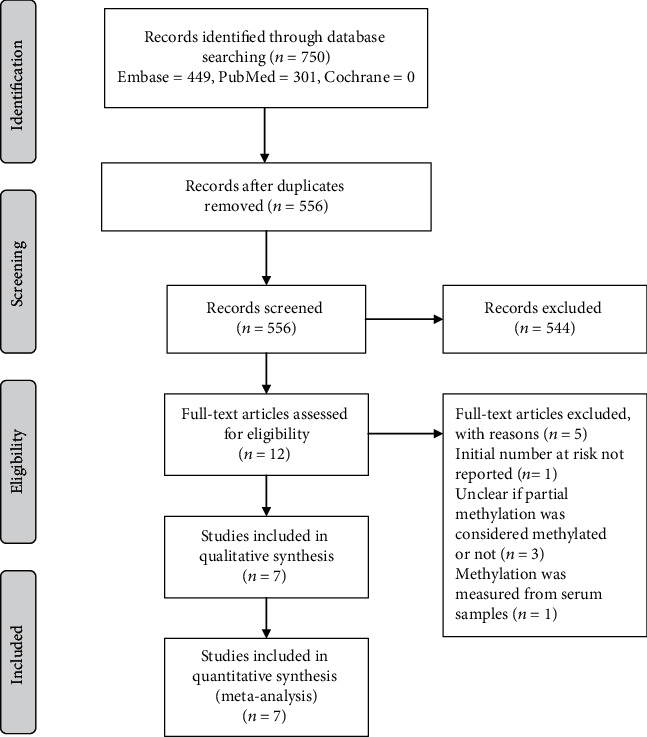
Summary of the search strategy performed to identify relevant studies to be included in the analysis.

We identified 750 potential papers from our search. The PRISMA (Preferred Reporting Items for Systematic Reviews and Meta-Analyses) statement was followed (Figure 1) There were seven included articles for which IPD for each gene was extracted. A summary of the IPD retrieved is displayed in Table 1. IPD is available in Supplementary Table 1.

### 3.1. RASSF1A

**Figure 2:**
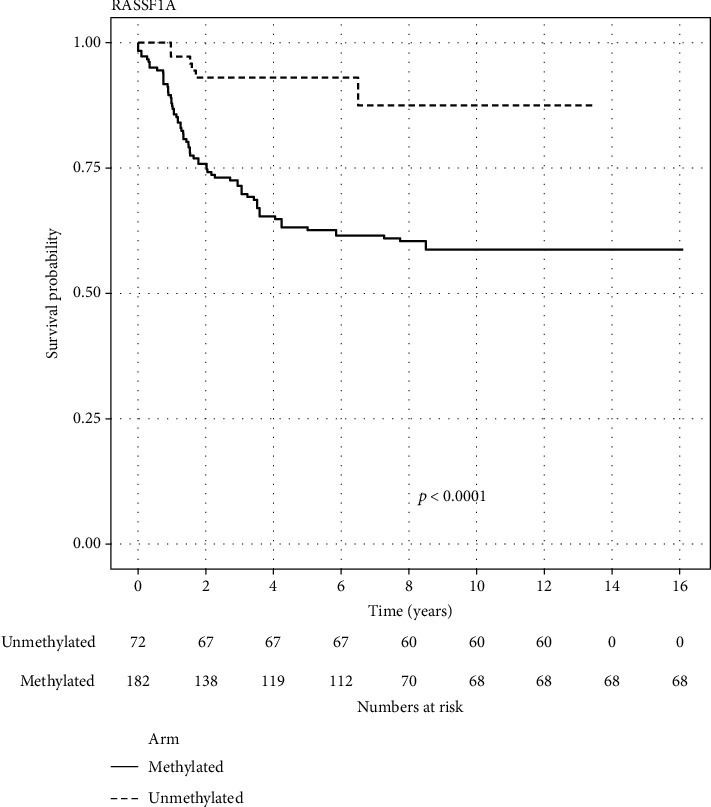
Reconstructed Kaplan–Meier graph of overall survival according to RASSF1A methylation using aggregated data.

Data on RASSF1A for 254 patients from three different studies were included. Out of which, 182 (71.65%) were methylated. The methylated arm had statistically significant worse OS than the unmethylated arm (log-rank test *p* value < 0.0001). 5-year survival probability for the methylated arm was 63.19% (95% CI 56.55-70.60), while for the unmethylated arm, it was 93.10% (95% CI 87.40–99.1).  Figure 2 displays the KM of the aggregated data for the RASSF1A gene. Adjusted HR for the methylated arm was 4.36 (95% CI 2.16–8.81).

### 3.2. DCR2

**Figure 3:**
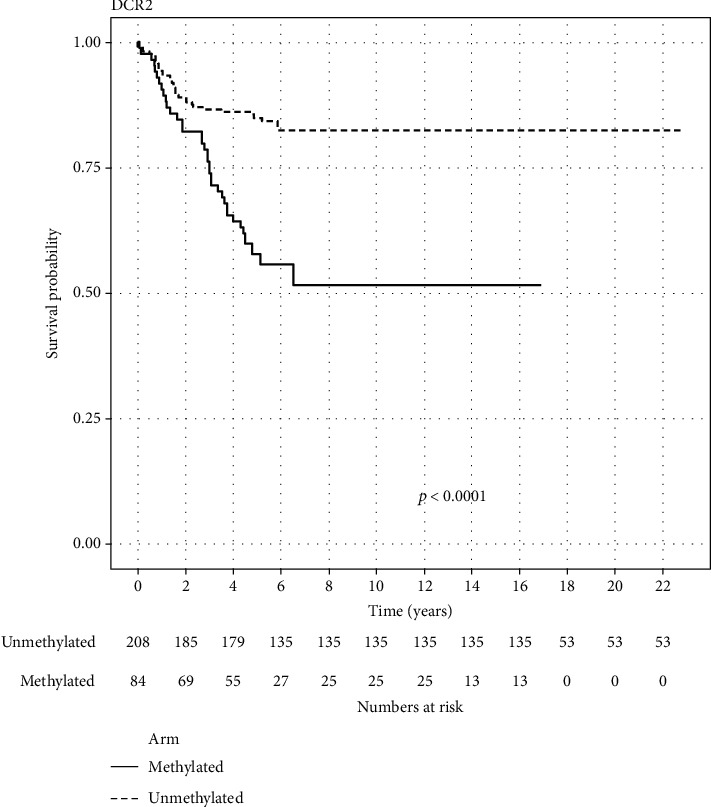
Reconstructed Kaplan–Meier graph of overall survival according to DCR2 methylation using aggregated data.

Data on DCR2 for 292 patients from three different studies were included. Out of which, 84 (28.77%) were methylated. The methylated arm had statistically significant worse OS than the unmethylated arm (log-rank test *p* value < 0.0001). 5-year survival probability for the methylated arm was 57.78% (95% CI 47.63-70.08), while for the unmethylated arm, it was 84.84% (95% CI 80.04-89.92).  Figure 3 displays the KM of the aggregated data for the DCR2 gene. Adjusted HR for the methylated arm was 3.53 (95% CI 2.20-5.65).

### 3.3. CASP8

**Figure 4:**
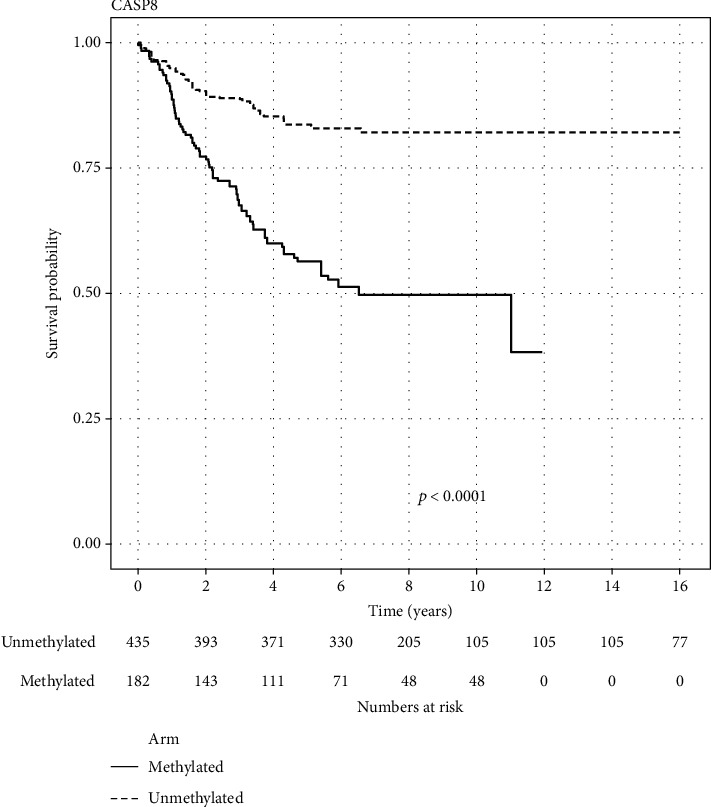
Reconstructed Kaplan–Meier graph of overall survival according to CASP8 methylation using aggregated data.

Data on CASP8 for 620 patients from five different studies were included. Out of which, 185 (29.84%) were methylated. The methylated arm had statistically significant worse OS than the unmethylated arm (log-rank test *p* value < 0.0001). 5-year survival probability for the methylated arm was 56.39% (95% CI 49.53-64.19), while for the unmethylated arm, it was 83.68% (95% CI 80.28-87.22).  Figure 4 displays the KM of the aggregated data for the CASP8 gene. Adjusted HR for the methylated arm was 4.66 (95% CI 3.42–6.35).

## 4. Discussion

Carcinogenesis is thought to be a result of interaction of genetic and epigenetic alterations [28, 29]. Epigenetic alterations, by definition, comprise mitotically and meiotically heritable changes in gene expression that are not caused by changes in the primary DNA sequence [30]. Perhaps, DNA methylation is the most extensively studied epigenetic alteration [28, 31]. DNA methylation is the addition of a methyl group 5 of the cytosine within the dinucleotide CpG. Hypermethylation of CpG islands, which are often present in gene promoters, leads to gene silencing. This has emerged as an important feature of both development and progression in many cancers [20, 22, 32].

Current risk classification of NB patients is based mainly on age of onset, disease stage at diagnosis, and MYCN amplification. Although this risk classification has been significantly improved in recent years, undertreatment or overtreatment will still occur for certain children. This results in suboptimal survival rates for some patients in the low-risk group and exposes some patients in the high-risk group to unnecessarily risk for potential long-term side effects of the toxic therapy. Therefore, it is important to have additional biomarkers included in the current risk classification. Only then, patients will receive the most appropriate therapy, without putting them at risk of under- or overtreatment [16, 24].

The neuroblastoma genome displays distinct patterns of DNA methylation which may have a clinicopathologic value and hence can be associated with different risk groups [33, 34]. RASSF1A, DCR2, and CASP8 are the most studied genes for correlation between their methylation and survival outcome. Most of these previous studies included few numbers of patients. In this study, we quantitatively reviewed the effect of DNA methylation of these three genes (RASSF1A, DCR2, and CASP8) on survival in NB patients. This is the first meta-analysis to combine the results of these studies aimed at showing their potential as a prognostic marker in NB patients. Reconstructed IPD was used in this study.

### 4.1. RASSF1A

RASSF1A (Ras association domain family protein 1 isoform A) was described by Dammann et al. as a Ras effector located at 3p21.3 [35, 36]. The RASSF1A gene encodes a protein like that of Ras effectors which exerts its function through a Ras signal transduction pathway. The RASSF1A induces growth arrest by inhibiting the accumulation of native cyclin D1 and preventing cells from passing through the retinoblastoma family cell cycle restriction point and entering the S phase [21, 24, 36, 37]. Therefore, loss of expression or altered expression through methylation of this gene has been associated with the pathogenesis of variety of cancers such as lung, breast, ovarian, and kidney cancers and pediatric tumors [36, 38–40].

In our study, RASSF1A was found methylated in 72% of NB patients; this was derived from the three studies included where methylation of RASSF1A ranged from 62% to 95% [21, 41, 42]. Our result is similar to previous studies not included in this analysis, which have found RASSF1A to be methylated in 50–100% of NB tumor specimens [24, 36, 43, 44]. Methylation of RASSF1A is less prevalent in serum samples compared to tumor samples. This was observed in one study comparing the RASSF1A methylation in serum with tumor samples revealing that RASSF1A was methylated in 17/68 (25%) in serum samples versus 64/68 (94%) in tumor samples of neuroblastoma patients [43]. Association between RASSF1A methylation and other known prognostic factors has been tested in many previous studies. One study reported that RASSF1A methylation was statistically significantly more prevalent in MYCN amplified tumors than in MYCN nonamplified tumors [44]. On the other hand, two studies (one of them is included in our meta-analysis) reported that there was no statistically significant difference in RASSF1A methylation prevalence according to MYCN amplification [21, 45]. Two studies (one of them is included in our meta-analysis) concluded that RASSF1A methylation in NB patients was statistically significantly more prevalent in the age group > 1 year, compared to the age group < 1 year [21, 38]. The study included in our analysis also concluded that RASSF1A methylation was statistically significantly correlated with the risk group, as RASSF1A methylation was more prevalent in the high-risk group [21]. Another study stratified neuroblastoma patients according to whether the tumor sample was from a primary or a relapsed tumor. This study found that RASSF1A was methylated in all relapsed tumors 17/17 (100%) and in 42/45 (93%) of primary tumors [44]. Further studies with a larger number of patients may be needed to confirm these associations between RASSF1A methylation and known risk factors.

In our study, 5-year survival probability for the RASSF1A methylated arm was 63% (95% CI 56.55-70.60), while for the unmethylated arm, it was 93.10% (95% CI 87.40–99.1) (Figure 2) (log-rank *p* value < 0.0001). Adjusted HR for the methylated arm was 4.36 (95% CI 2.16–8.81). These results are consistent with the results in some of the included studies in our meta-analysis [21, 42], while two other studies (one of them is included in our meta-analysis) failed to demonstrate statistical significance perhaps due to small sample size or short follow-up duration [41, 44]. The association between RASSF1A methylation and poor prognosis may be explained by its association with known poor prognostic factors such as older age, MYCN amplification, and high-risk group as previously discussed. This may not be entirely true as one study showed that patients with stage 4 NB and older than 1 year having tumors with a high percentage of RASSF1A methylation (>70%) had a significantly worse outcome than patients with similar prognostic criteria and a low percentage of RASSF1A methylation (5-year OS 19% vs. 56%, respectively) [46]. Since RASSF1A may be correlated with other risk factors, multivariate analysis will be necessary to determine if RASSF1A is an independent prognostic factor. One study included in our meta-analysis conducted a multivariate analysis concluding that RASSF1A was an independent prognostic factor [42]. Interestingly, another study conducted multivariate analysis of survival including methylation of RASSF1A in serum as a prognostic variable. The hazard ratio of this analysis was 2.4 (95% CI, 0.6–9.2); although this association did not reach statistical significance, these findings show that the methylation status of RASSF1A in the serum of patients with neuroblastoma may have the potential to become a prognostic predictor of outcome. This can be fostered by the results of the univariate analysis which showed that the influence of serum RASSF1A methylation on prognosis was comparable with that of the currently most reliable marker, MYCN amplification [43]. Most of other studies did not perform a multivariate analysis due to small sample size of included patients. Therefore, future studies will need to be performed with larger sample size to confirm our findings and to demonstrate if the RASSF1A can be used as an independent prognostic factor.

Some studies showed that RASSF1A expression can be restored in cell lines after treatment with the demethylating agent 5-Aza-dC [21, 47]. This may be considered a potential agent to be used in combination with chemotherapeutic drugs to treat hypermethylated RASSF1A neuroblastoma [48].

### 4.2. DCR2

DCR2 (decoy receptor 2) (TNRSF10D) is a tumor necrosis factor-*α* receptor family gene that is located on 8p21 [44, 49, 50]. The exact role that DCR2 plays in tumorigenesis remains unclear, as inactivation of DCR2 should have antitumor effects by sensitizing the cells to TRAIL-induced apoptosis [24]. However, the downregulation of DCR2 by promoter methylation is reported in various types of cancer [42, 49, 50]. One of the theories explaining this phenomenon is that it could be considered part of an inefficient defense mechanism activated to inhibit tumor cell growth [23, 44].

In our study, DCR2 was found methylated in 29% of NB patients; this was derived from the three studies included where methylation of DCR2 ranged from 21% to 44% [23, 42, 49]. Our result is similar to previous studies not included in this analysis, which have found DCR2 to be methylated in 25%–42% of NB tumor specimens [41, 44]. One of the included studies in our analysis studied DCR2 methylation in serum and found that it was significantly correlated with DCR2 methylation in tumors (*r* = 0.67) [49]. Association between DCR2 methylation and other known prognostic factors has been tested in many previous studies. The frequency of DCR2 methylation was statistically significantly higher in high-risk groups compared to low-risk groups in one of the included studies in our meta-analysis [49]. This study further analyzed the frequency of DCR2 methylation focusing on patients with MYCN nonamplified tumors only. The prevalence of DCR2 methylation in the high-risk group was still statistically significantly higher than that in the low-risk group. Moreover, the frequency of DCR2 methylation between patients with MYCN amplified tumors was statistically significantly higher than that in patients with MYCN nonamplified tumors [49]. However, two other studies (one of them is included in our meta-analysis) concluded that DCR2 methylation is independent of MYCN amplification [42, 44]. Another study stratified neuroblastoma patients according to whether the tumor sample was from a primary or a relapsed tumor. This study found that DCR2 was methylated in all 29% of relapsed tumors and in 25% of primary tumors, concluding that there is no change in the methylation profile at relapse [41]. Further studies with a larger number of patients may be needed to confirm these associations between DCR2 methylation and known risk factors.

In our study, 5-year survival probability for the DCR2 methylated arm was 57.78% (95% CI 47.63-70.08), while for the unmethylated arm, it was 84.84% (95% CI 80.04-89.92) (Figure 3) (log-rank *p* value < 0.0001). Adjusted HR for the methylated arm was 3.53 (95% CI 2.20-5.65). These results are consistent with the results in all of the studies included in our meta-analysis and other previous studies [23, 42, 44, 49], while only one study failed to prove a statistically significant difference in OS between methylated and unmethylated DCR2 [41]. Two studies (one of them is included in our meta-analysis) analyzed the effect of DCR2 methylation in MYCN nonamplified tumors on overall survival. The first showed a trend for the negative prognostic effect of DCR2 methylation, failing to reach statistical significance mainly because of the small sample size in this cohort (17 patients) [44], while the included study was able to demonstrate statistical significance where patients with DCR2 methylation had a worse outcome than patients with unmethylated DCR2 (56% versus 96%, respectively; *p* < 0.001) [49]. The same study tried to evaluate if an increase in serum DCR2 methylation can be used as an indicator to relapse. They measured serum DCR2 methylation in the clinical course of five patients. In two patients who achieved complete remission, serum DCR2 methylation decreased to an undetectable level, while in three patients who had recurrence after remission, the serum DCR2 methylation first decreased to an undetectable level and then increased again at the time of diagnosis [49]. In multivariate analysis to determine the prognostic effect of DCR2 methylation, two studies (one of them is included in our meta-analysis) failed to show that DCR2 methylation was a statistically significant independent prognostic factor probably due to the small sample size and large number of prognostic factors included in the model [42, 44], although one of these studies demonstrated that the combination of DCR2 and RASSF1A methylation was statistically significant in the multivariate model with RR 3.79 (95% CI 1.01–14.22) [44]. The included study also found that DCR2 methylation was correlated with a poor outcome in children with a triploid, not diploid, tumor [42].

### 4.3. CASP8

CASP8 (located on human chromosome 2 band q33) methylation was first reported in neuroblastoma tumors nearly 18 years ago [51]. The CASP8 gene encodes a member of the cysteine-aspartic acid protease (caspase) family, which exerts its function inducing a death signaling complex through the Fas ligand pathway. The N-terminal FADD-like death effector domain of this protein suggests that it may interact with the Fas-interacting protein FADD [24, 36, 51–53]. Therefore, loss of expression or altered expression through methylation of this gene has been associated with the pathogenesis of variety of cancers such as neuroblastoma, medulloblastoma, lung cancer, and colorectal carcinomas [54–56].

In our study, CASP8 was found methylated in 30% of NB patients; this was derived from the five studies included where methylation of CASP8 ranged from 19% to 56% [23, 41, 42, 57, 58]. Our result is similar to previous studies not included in this analysis, which have found CASP8 to be methylated in 14%–62% of NB tumor specimens [36, 45, 51, 59–62]. There was only one study, which included 11 patients with stage 4 NB and found that CASP8 is methylated in 91% of tumor samples. This study also measured CASP8 methylation in both tumors and bone marrow samples. They found that CASP8 methylation was found in 55% of bone marrow samples [63].

Association between CASP8 methylation and known risk factors such as MYCN amplification, tumor stage, and age at diagnosis was studied in previous studies. There is a wide controversy in literature regarding the correlation between CASP8 methylation and MYCN amplification. Five studies (two of them are included in our meta-analysis) found that there was statistically significant correlation with CASP8 methylation and MYCN amplification as CASP8 methylation was more prevalent in MYCN amplified tumors [42, 45, 51, 57, 62], while nine other studies (two of them are included in our meta-analysis) failed to prove this association concluding that CASP8 is not correlated with MYCN amplification [23, 36, 41, 44, 45, 61, 64–66]. One meta-analysis was done to test the significance of this correlation, combining the results of three individual studies, which failed to demonstrate the statistical significance. The result was statistically significant with CASP8 methylation in 66% of MYCN amplified tumors versus 36% in MYCN nonamplified samples [45]. Similar controversy exists for the correlation between CASP8 and the stage of the disease. Three studies (two of them are included in our meta-analysis) were able to prove that CASP8 methylation was more prevalent in advanced stages [41, 57, 62], while three other studies did not reach statistical significance for this correlation [45, 51, 65]. One study found that there was no statistically significant correlation between CASP8 methylation and age at diagnosis [65]. One study found that there was statistically significant correlation between RASSF1A methylation and CASP8 methylation [60]. Further studies with a larger number of patients may be needed to confirm these associations between CASP8 methylation and known risk factors.

In our study, 5-year survival probability for the CASP8 methylated arm was 56.39% (95% CI 49.53-64.19), while for the unmethylated arm, it was 83.68% (95% CI 80.28-87.22) (Figure 4) (log-rank *p* value < 0.0001). Adjusted HR for the methylated arm was 4.66 (95% CI 3.42–6.35). These results are consistent with the results in the included studies in our analysis and other previous studies [23, 42, 45, 57, 58, 65]. Except for one study included in our meta-analysis which failed to prove a statistically significant difference between both arms, there was a numerical difference favoring the same trend [41]. In multivariate analysis to determine the prognostic effect of CASP8 methylation, two studies (one of them is included in our meta-analysis) showed that CASP8 methylation was an independent prognostic variable [57, 65]. The study included in our meta-analysis analyzed more genes; then, they grouped genes according to their cellular function to evaluate the effect of each group on the survival rates. Methylation of the apoptosis-related genes was the only group which showed a statistically significant effect on survival [57]. Another study included in our meta-analysis recruited patients from Germany and Japan. CASP8 methylation had a similar effect on survival in both populations [58]. In another study, the CASP8 protein expression effect on survival was evaluated in 140 NB patients instead of CASP8 methylation. Surprisingly, CASP8 protein expression did not have an effect on survival [59].

Restoration of CASP8 expression in cell lines has been shown in previous studies to be necessary to restore TRAIL-mediated cell death [67–69]. IFN-*γ* showed clinical evidence for restoration of CASP8 expression in NB patients in two previous small studies. However, there was no clinical correlation in these two studies [70, 71]. Another study examined the combination of 5-dAzaC with IFN-*γ* at relatively low individual drug doses. This combination was found to have a synergistic effect in CASP8 gene activation [72]. The molecular basis of this synergistic interaction of 5-dAzaC and IFN-*γ* may be explained by the different mechanisms of action of each agent to upregulate caspase-8. 5-dAzaC is a demethylating agent that reverses hypermethylation of a gene regulatory region of the caspase-8 gene [73], whereas IFN-*γ* enhances CASP8 levels through transcriptional activation of CASP8 through a Stat-1/IRF1-dependent pathway without altering the methylation status of the caspase-8 gene [74]. Restoration of CASP8 expression in deficient neuroblastoma cells suppressed their metastases [75].

Our meta-analysis used IPD, as combining results of a survival time outcome using conventional methods such as pooling analysis from hazard ratios or time point outcomes is definitely challenging. One reason for that is that endpoints may be reported using different methods, as some studies may report HR while others may report median survival. Another reason would be that combining HRs from different studies may be subjected to bias, as these studies should check that the proportional hazard assumption is fulfilled. This assumption is seldom checked in most trials [76]. Several methods have been proposed earlier to extract data from published KM in order to carry out a meta-analysis. But most of these methods did not focus on the reconstruction of the KM or the life table data [77, 78]. Therefore, the used algorithm developed by Guyot et al. was deemed the most appropriate for the purpose of this analysis [26]. This algorithm uses coordinates from KM extracted by digital software and the information on the initial number at risk and at different time points along with the number of events if available. The mean absolute error if all the information is available is certainly lower than that if only the initial number at risk is available. In studies included in our analysis, the number at risk at different time points and the number of events were not reported. The mean absolute error for determining survival probability if these data were not available is reported to be 0.328% (95% CI 0.031; 2.233), while for HR, the mean absolute error is higher 0.198 (95% CI 0.021; 1.556) [26]. Therefore, this is one of the limitations of our study, and thus, we encourage future studies with time-to-event outcomes to report numbers at risk at different points on the KM along with the number of events.

Other limitation of our study is that we pooled data from different populations over different covariates. We tried decreasing the impact of this by using multivariate Cox regression adjustment including the source of data as a covariate to adjust for this bias. Although the trend of results for the different papers included was generally similar, interpretation of our endpoints must be done with caution. In addition to that, our analysis used reconstructed IPD according to the methylation status only; as a result, we did not have any data for other risk factors. Hence, we were unable to perform multivariate analysis for the methylation of the studied genes on survival taking into account other known risk factors (e.g., MYCN amplification). Therefore, future large studies are needed to collect data about methylation of these genes along with other risk factors.

In conclusion, our results indicate that in NB patients, RASSF1A, DCR2, and CASP8 methylation is associated with poor prognosis. The fact that methylation of these genes may be reversible makes them a potential therapeutic target [79]. In addition, epigenetic alterations of these genes may be used as a marker of the disease. Large prospective studies will be necessary to confirm definitive correlation between methylation of these genes and survival outcome taking into account all other known risk factors.

## Figures and Tables

**Table 1:** Summary of included studies in the analysis.

PMID	Author	Country of origin	CASP8			DCR2			RASSF1		
			Methylated	Unmethylated	Total	Methylated	Unmethylated	Total	Methylated	Unmethylated	Total
15623630	Yang et al. [21]	USA	—	—	—	—	—	—	39	17	56
17545522	Yang et al. [23]	USA	39	31	70	31	39	70	—	—	—
17570703	Michalowski et al. [48]	France	23	39	62	—	—	—	59	3	62
18980997	Yagyu et al. [49]	Japan	—	—	—	24	62	86	—	—	—
21104989	Grau et al. [63]	Spain	31	29	60	—	—	—	—	—	—
23619990	Asada et al. [58]	Germany-Japan	56	236	292	—	—	—	—	—	—
24680815	Haruta et al. [42]	Japan	36	100	136	29	107	136	84	52	136
Grand total			185	435	620	84	208	292	182	72	254

## Supplementary Materials

Supplementary Materials Supplementary Table 1: details of individual patient data retrieved.
