# Supplementary material for: Association of RASSF1A, DCR2, and CASP8 Methylation with Survival in Neuroblastoma: A Pooled Analysis Using Reconstructed Individual Patient Data
Source: Biomed Res Int. 2020 Dec 15;2020:7390473. doi: 10.1155/2020/7390473 (PMC7755470; doi:10.1155/2020/7390473)
Supplement: Supplementary Materials — Supplementary Table 1: details of individual patient data retrieved. [file 7390473.f1.pdf]

| ID | Time | Status | Arm | Paper (PMID) | Gene |
|---|---|---|---|---|---|
| 1106 | 0.0721 | 1 | Methylated | 17545522 | CASP8 |
| 1103 | 0.0734 | 1 | Methylated | 17545522 | DCR2 |
| 280 | 0.08 | 1 | Methylated | 17545522 | CASP8 |
| 1100 | 0.089 | 1 | Methylated | 23619990 | CASP8 |
| 270 | 0.102 | 1 | Methylated | 15623630 | RASSF1 |
| 1105 | 0.102 | 1 | Methylated | 15623630 | RASSF1 |
| 250 | 0.135 | 1 | Methylated | 17545522 | DCR2 |
| 123 | 0.18 | 1 | Unmethylated | 23619990 | CASP8 |
| 210 | 0.18 | 1 | Unmethylated | 23619990 | CASP8 |
| 1108 | 0.236 | 1 | Unmethylated | 17570703 | CASP8 |
| 2100 | 0.236 | 1 | Unmethylated | 17570703 | CASP8 |
| 1109 | 0.267 | 1 | Methylated | 17570703 | RASSF1 |
| 140 | 0.321 | 1 | Methylated | 17570703 | CASP8 |
| 220 | 0.321 | 1 | Methylated | 17570703 | CASP8 |
| 347 | 0.344 | 1 | Methylated | 17570703 | RASSF1 |
| 2101 | 0.344 | 1 | Methylated | 17570703 | RASSF1 |
| 336 | 0.384 | 1 | Methylated | 17545522 | CASP8 |
| 1 | 0.39 | 1 | Unmethylated | 23619990 | CASP8 |
| 2 | 0.39 | 1 | Unmethylated | 23619990 | CASP8 |
| 3 | 0.39 | 1 | Unmethylated | 23619990 | CASP8 |
| 4 | 0.39 | 1 | Unmethylated | 23619990 | CASP8 |
| 5 | 0.39 | 1 | Unmethylated | 23619990 | CASP8 |
| 310 | 0.45 | 1 | Unmethylated | 23619990 | CASP8 |
| 410 | 0.45 | 1 | Unmethylated | 23619990 | CASP8 |
| 1107 | 0.489 | 1 | Unmethylated | 17545522 | DCR2 |
| 334 | 0.564 | 1 | Methylated | 15623630 | RASSF1 |
| 330 | 0.576 | 1 | Methylated | 17545522 | DCR2 |
| 434 | 0.589 | 1 | Methylated | 17545522 | CASP8 |
| 240 | 0.63 | 1 | Methylated | 23619990 | CASP8 |
| 328 | 0.63 | 1 | Methylated | 23619990 | CASP8 |
| 149 | 0.714 | 1 | Methylated | 18980997 | DCR2 |
| 160 | 0.72 | 1 | Methylated | 23619990 | CASP8 |
| 319 | 0.743 | 1 | Methylated | 17570703 | CASP8 |
| 437 | 0.744 | 1 | Methylated | 17570703 | RASSF1 |
| 260 | 0.823 | 1 | Methylated | 21104989 | CASP8 |
| 1104 | 0.823 | 1 | Methylated | 21104989 | CASP8 |
| 1135 | 0.857 | 1 | Unmethylated | 18980997 | DCR2 |
| 6 | 0.86 | 1 | Unmethylated | 23619990 | CASP8 |
| 433 | 0.871 | 1 | Methylated | 15623630 | RASSF1 |
| 535 | 0.897 | 1 | Methylated | 17570703 | RASSF1 |
| 229 | 0.92 | 1 | Methylated | 23619990 | CASP8 |
| 7 | 0.92 | 1 | Unmethylated | 23619990 | CASP8 |
| 8 | 0.92 | 1 | Unmethylated | 23619990 | CASP8 |
| 530 | 0.973 | 1 | Methylated | 15623630 | RASSF1 |
| 419 | 0.98 | 1 | Methylated | 17570703 | CASP8 |
| 324 | 0.99 | 1 | Methylated | 23619990 | CASP8 |

| | | | | | |
|---|---|---|---|---|---|
| 424 | 0.99 | 1 | Methylated | 23619990 | CASP8 |
| 225 | 1 | 1 | Methylated | 18980997 | DCR2 |
| 634 | 1.01 | 1 | Methylated | 17570703 | RASSF1 |
| 629 | 1.02 | 1 | Methylated | 15623630 | RASSF1 |
| 519 | 1.06 | 1 | Methylated | 17570703 | CASP8 |
| 532 | 1.07 | 1 | Methylated | 17545522 | CASP8 |
| 429 | 1.07 | 1 | Methylated | 17545522 | DCR2 |
| 618 | 1.08 | 1 | Methylated | 17570703 | CASP8 |
| 427 | 1.1 | 1 | Methylated | 23619990 | CASP8 |
| 9 | 1.1 | 1 | Unmethylated | 23619990 | CASP8 |
| 510 | 1.1 | 1 | Unmethylated | 23619990 | CASP8 |
| 610 | 1.1 | 1 | Unmethylated | 23619990 | CASP8 |
| 718 | 1.11 | 1 | Methylated | 17570703 | CASP8 |
| 729 | 1.13 | 1 | Methylated | 15623630 | RASSF1 |
| 734 | 1.18 | 1 | Methylated | 17570703 | RASSF1 |
| 834 | 1.18 | 1 | Methylated | 17570703 | RASSF1 |
| 523 | 1.2 | 1 | Methylated | 23619990 | CASP8 |
| 320 | 1.21 | 1 | Methylated | 18980997 | DCR2 |
| 340 | 1.23 | 1 | Unmethylated | 17570703 | CASP8 |
| 436 | 1.23 | 1 | Unmethylated | 17570703 | CASP8 |
| 818 | 1.25 | 1 | Methylated | 17570703 | CASP8 |
| 934 | 1.26 | 1 | Methylated | 17570703 | RASSF1 |
| 1034 | 1.26 | 1 | Methylated | 17570703 | RASSF1 |
| 1134 | 1.28 | 1 | Methylated | 17570703 | RASSF1 |
| 526 | 1.3 | 1 | Methylated | 23619990 | CASP8 |
| 534 | 1.32 | 1 | Unmethylated | 17570703 | CASP8 |
| 829 | 1.33 | 1 | Methylated | 15623630 | RASSF1 |
| 333 | 1.33 | 1 | Methylated | 21104989 | CASP8 |
| 1228 | 1.34 | 1 | Methylated | 17570703 | RASSF1 |
| 1324 | 1.34 | 1 | Methylated | 17570703 | RASSF1 |
| 420 | 1.36 | 1 | Methylated | 18980997 | DCR2 |
| 349 | 1.36 | 1 | Unmethylated | 18980997 | DCR2 |
| 2102 | 1.36 | 1 | Unmethylated | 18980997 | DCR2 |
| 633 | 1.4 | 1 | Unmethylated | 17570703 | CASP8 |
| 733 | 1.4 | 1 | Unmethylated | 17570703 | CASP8 |
| 622 | 1.4 | 1 | Methylated | 23619990 | CASP8 |
| 10 | 1.4 | 1 | Unmethylated | 23619990 | CASP8 |
| 1423 | 1.43 | 1 | Methylated | 17570703 | RASSF1 |
| 439 | 1.43 | 1 | Unmethylated | 18980997 | DCR2 |
| 929 | 1.49 | 1 | Methylated | 15623630 | RASSF1 |
| 833 | 1.49 | 1 | Unmethylated | 17570703 | CASP8 |
| 933 | 1.49 | 1 | Unmethylated | 17570703 | CASP8 |
| 537 | 1.5 | 1 | Unmethylated | 18980997 | DCR2 |
| 1522 | 1.53 | 1 | Methylated | 17570703 | RASSF1 |
| 1622 | 1.53 | 1 | Methylated | 17570703 | RASSF1 |
| 1029 | 1.54 | 1 | Methylated | 15623630 | RASSF1 |
| 127 | 1.54 | 1 | Unmethylated | 15623630 | RASSF1 |

| | | | | | |
|---|---|---|---|---|---|
| 635 | 1.57 | 1 | Unmethylated | 18980997 | DCR2 |
| 625 | 1.6 | 1 | Methylated | 23619990 | CASP8 |
| 725 | 1.6 | 1 | Methylated | 23619990 | CASP8 |
| 11 | 1.6 | 1 | Unmethylated | 23619990 | CASP8 |
| 12 | 1.6 | 1 | Unmethylated | 23619990 | CASP8 |
| 13 | 1.6 | 1 | Unmethylated | 23619990 | CASP8 |
| 14 | 1.6 | 1 | Unmethylated | 23619990 | CASP8 |
| 1129 | 1.64 | 1 | Methylated | 15623630 | RASSF1 |
| 630 | 1.65 | 1 | Methylated | 17545522 | CASP8 |
| 528 | 1.65 | 1 | Methylated | 17545522 | DCR2 |
| 825 | 1.7 | 1 | Methylated | 23619990 | CASP8 |
| 710 | 1.7 | 1 | Unmethylated | 23619990 | CASP8 |
| 735 | 1.71 | 1 | Unmethylated | 18980997 | DCR2 |
| 722 | 1.8 | 1 | Methylated | 23619990 | CASP8 |
| 170 | 1.81 | 1 | Unmethylated | 21104989 | CASP8 |
| 822 | 2 | 1 | Methylated | 23619990 | CASP8 |
| 15 | 2 | 1 | Unmethylated | 23619990 | CASP8 |
| 16 | 2 | 1 | Unmethylated | 23619990 | CASP8 |
| 810 | 2 | 1 | Unmethylated | 23619990 | CASP8 |
| 910 | 2 | 1 | Unmethylated | 23619990 | CASP8 |
| 1010 | 2 | 1 | Unmethylated | 23619990 | CASP8 |
| 1224 | 2.05 | 1 | Methylated | 15623630 | RASSF1 |
| 430 | 2.07 | 1 | Methylated | 21104989 | CASP8 |
| 922 | 2.1 | 1 | Methylated | 23619990 | CASP8 |
| 925 | 2.2 | 1 | Methylated | 23619990 | CASP8 |
| 1022 | 2.2 | 1 | Methylated | 23619990 | CASP8 |
| 1025 | 2.2 | 1 | Methylated | 23619990 | CASP8 |
| 17 | 2.4 | 1 | Unmethylated | 23619990 | CASP8 |
| 627 | 2.69 | 1 | Methylated | 17545522 | DCR2 |
| 727 | 2.69 | 1 | Methylated | 17545522 | DCR2 |
| 730 | 2.7 | 1 | Methylated | 17545522 | CASP8 |
| 830 | 2.7 | 1 | Methylated | 17545522 | CASP8 |
| 1320 | 2.72 | 1 | Methylated | 15623630 | RASSF1 |
| 520 | 2.79 | 1 | Methylated | 18980997 | DCR2 |
| 529 | 2.89 | 1 | Methylated | 21104989 | CASP8 |
| 1125 | 2.9 | 1 | Methylated | 23619990 | CASP8 |
| 1220 | 2.9 | 1 | Methylated | 23619990 | CASP8 |
| 628 | 2.97 | 1 | Methylated | 21104989 | CASP8 |
| 728 | 2.97 | 1 | Methylated | 21104989 | CASP8 |
| 619 | 3 | 1 | Methylated | 18980997 | DCR2 |
| 18 | 3 | 1 | Unmethylated | 23619990 | CASP8 |
| 1122 | 3.2 | 1 | Methylated | 23619990 | CASP8 |
| 1217 | 3.2 | 1 | Methylated | 23619990 | CASP8 |
| 1419 | 3.23 | 1 | Methylated | 15623630 | RASSF1 |
| 1316 | 3.3 | 1 | Methylated | 23619990 | CASP8 |
| 1415 | 3.3 | 1 | Methylated | 23619990 | CASP8 |
| 124 | 3.3 | 1 | Unmethylated | 23619990 | CASP8 |

| | | | | | |
|---|---|---|---|---|---|
| 131 | 3.3 | 1 | Unmethylated | 23619990 | CASP8 |
| 1110 | 3.3 | 1 | Unmethylated | 23619990 | CASP8 |
| 719 | 3.36 | 1 | Methylated | 18980997 | DCR2 |
| 828 | 3.38 | 1 | Methylated | 21104989 | CASP8 |
| 918 | 3.4 | 1 | Methylated | 17570703 | CASP8 |
| 1018 | 3.4 | 1 | Methylated | 17570703 | CASP8 |
| 19 | 3.4 | 1 | Unmethylated | 23619990 | CASP8 |
| 20 | 3.4 | 1 | Unmethylated | 23619990 | CASP8 |
| 21 | 3.4 | 1 | Unmethylated | 23619990 | CASP8 |
| 1721 | 3.42 | 1 | Methylated | 17570703 | RASSF1 |
| 835 | 3.5 | 1 | Unmethylated | 18980997 | DCR2 |
| 1518 | 3.59 | 1 | Methylated | 15623630 | RASSF1 |
| 1819 | 3.59 | 1 | Methylated | 17570703 | RASSF1 |
| 1918 | 3.59 | 1 | Methylated | 17570703 | RASSF1 |
| 141 | 3.6 | 1 | Unmethylated | 23619990 | CASP8 |
| 151 | 3.6 | 1 | Unmethylated | 23619990 | CASP8 |
| 161 | 3.6 | 1 | Unmethylated | 23619990 | CASP8 |
| 22 | 3.7 | 1 | Unmethylated | 23619990 | CASP8 |
| 930 | 3.74 | 1 | Methylated | 17545522 | CASP8 |
| 1030 | 3.74 | 1 | Methylated | 17545522 | CASP8 |
| 1130 | 3.74 | 1 | Methylated | 17545522 | CASP8 |
| 827 | 3.75 | 1 | Methylated | 17545522 | DCR2 |
| 927 | 3.75 | 1 | Methylated | 17545522 | DCR2 |
| 1313 | 3.8 | 1 | Methylated | 23619990 | CASP8 |
| 1412 | 3.8 | 1 | Methylated | 23619990 | CASP8 |
| 1618 | 4.05 | 1 | Methylated | 15623630 | RASSF1 |
| 309 | 4.23 | 0 | Methylated | 17545522 | CASP8 |
| 337 | 4.23 | 0 | Methylated | 17545522 | CASP8 |
| 344 | 4.23 | 0 | Methylated | 17545522 | CASP8 |
| 354 | 4.23 | 0 | Methylated | 17545522 | CASP8 |
| 364 | 4.23 | 0 | Methylated | 17545522 | CASP8 |
| 374 | 4.23 | 0 | Methylated | 17545522 | CASP8 |
| 384 | 4.23 | 0 | Methylated | 17545522 | CASP8 |
| 394 | 4.23 | 0 | Methylated | 17545522 | CASP8 |
| 1225 | 4.23 | 0 | Methylated | 17545522 | CASP8 |
| 1321 | 4.23 | 0 | Methylated | 17545522 | CASP8 |
| 1420 | 4.23 | 0 | Methylated | 17545522 | CASP8 |
| 1519 | 4.23 | 0 | Methylated | 17545522 | CASP8 |
| 1619 | 4.23 | 0 | Methylated | 17545522 | CASP8 |
| 1718 | 4.23 | 0 | Methylated | 17545522 | CASP8 |
| 1816 | 4.23 | 0 | Methylated | 17545522 | CASP8 |
| 1915 | 4.23 | 0 | Methylated | 17545522 | CASP8 |
| 2015 | 4.23 | 0 | Methylated | 17545522 | CASP8 |
| 2121 | 4.23 | 0 | Methylated | 17545522 | CASP8 |
| 2218 | 4.23 | 0 | Methylated | 17545522 | CASP8 |
| 2316 | 4.23 | 0 | Methylated | 17545522 | CASP8 |
| 2414 | 4.23 | 0 | Methylated | 17545522 | CASP8 |

| | | | | | |
|---|---|---|---|---|---|
| 2512 | 4.23 | 0 | Methylated | 17545522 | CASP8 |
| 2611 | 4.23 | 0 | Methylated | 17545522 | CASP8 |
| 2710 | 4.23 | 0 | Methylated | 17545522 | CASP8 |
| 2810 | 4.23 | 0 | Methylated | 17545522 | CASP8 |
| 2910 | 4.23 | 0 | Methylated | 17545522 | CASP8 |
| 3114 | 4.23 | 0 | Methylated | 17545522 | CASP8 |
| 3211 | 4.23 | 0 | Methylated | 17545522 | CASP8 |
| 1514 | 4.3 | 1 | Methylated | 23619990 | CASP8 |
| 1614 | 4.3 | 1 | Methylated | 23619990 | CASP8 |
| 171 | 4.3 | 1 | Unmethylated | 23619990 | CASP8 |
| 181 | 4.3 | 1 | Unmethylated | 23619990 | CASP8 |
| 191 | 4.3 | 1 | Unmethylated | 23619990 | CASP8 |
| 201 | 4.3 | 1 | Unmethylated | 23619990 | CASP8 |
| 211 | 4.3 | 1 | Unmethylated | 23619990 | CASP8 |
| 819 | 4.43 | 1 | Methylated | 18980997 | DCR2 |
| 259 | 4.48 | 0 | Methylated | 17545522 | DCR2 |
| 268 | 4.48 | 0 | Methylated | 17545522 | DCR2 |
| 277 | 4.48 | 0 | Methylated | 17545522 | DCR2 |
| 287 | 4.48 | 0 | Methylated | 17545522 | DCR2 |
| 297 | 4.48 | 0 | Methylated | 17545522 | DCR2 |
| 306 | 4.48 | 0 | Methylated | 17545522 | DCR2 |
| 1027 | 4.48 | 0 | Methylated | 17545522 | DCR2 |
| 1127 | 4.48 | 0 | Methylated | 17545522 | DCR2 |
| 1222 | 4.48 | 0 | Methylated | 17545522 | DCR2 |
| 1318 | 4.48 | 0 | Methylated | 17545522 | DCR2 |
| 1417 | 4.48 | 0 | Methylated | 17545522 | DCR2 |
| 1516 | 4.48 | 0 | Methylated | 17545522 | DCR2 |
| 1616 | 4.48 | 0 | Methylated | 17545522 | DCR2 |
| 1715 | 4.48 | 0 | Methylated | 17545522 | DCR2 |
| 1813 | 4.48 | 0 | Methylated | 17545522 | DCR2 |
| 1912 | 4.48 | 0 | Methylated | 17545522 | DCR2 |
| 2012 | 4.48 | 0 | Methylated | 17545522 | DCR2 |
| 2118 | 4.48 | 0 | Methylated | 17545522 | DCR2 |
| 2215 | 4.48 | 0 | Methylated | 17545522 | DCR2 |
| 2313 | 4.48 | 0 | Methylated | 17545522 | DCR2 |
| 2411 | 4.48 | 0 | Methylated | 17545522 | DCR2 |
| 3111 | 4.48 | 0 | Methylated | 17545522 | DCR2 |
| 290 | 4.48 | 0 | Unmethylated | 17545522 | DCR2 |
| 338 | 4.48 | 0 | Unmethylated | 17545522 | DCR2 |
| 339 | 4.48 | 0 | Unmethylated | 17545522 | DCR2 |
| 345 | 4.48 | 0 | Unmethylated | 17545522 | DCR2 |
| 355 | 4.48 | 0 | Unmethylated | 17545522 | DCR2 |
| 365 | 4.48 | 0 | Unmethylated | 17545522 | DCR2 |
| 375 | 4.48 | 0 | Unmethylated | 17545522 | DCR2 |
| 385 | 4.48 | 0 | Unmethylated | 17545522 | DCR2 |
| 395 | 4.48 | 0 | Unmethylated | 17545522 | DCR2 |
| 435 | 4.48 | 0 | Unmethylated | 17545522 | DCR2 |

| | | | | | |
|---|---|---|---|---|---|
| 533 | 4.48 | 0 | Unmethylated | 17545522 | DCR2 |
| 632 | 4.48 | 0 | Unmethylated | 17545522 | DCR2 |
| 732 | 4.48 | 0 | Unmethylated | 17545522 | DCR2 |
| 832 | 4.48 | 0 | Unmethylated | 17545522 | DCR2 |
| 932 | 4.48 | 0 | Unmethylated | 17545522 | DCR2 |
| 1032 | 4.48 | 0 | Unmethylated | 17545522 | DCR2 |
| 1132 | 4.48 | 0 | Unmethylated | 17545522 | DCR2 |
| 1226 | 4.48 | 0 | Unmethylated | 17545522 | DCR2 |
| 1322 | 4.48 | 0 | Unmethylated | 17545522 | DCR2 |
| 1421 | 4.48 | 0 | Unmethylated | 17545522 | DCR2 |
| 1520 | 4.48 | 0 | Unmethylated | 17545522 | DCR2 |
| 1620 | 4.48 | 0 | Unmethylated | 17545522 | DCR2 |
| 1719 | 4.48 | 0 | Unmethylated | 17545522 | DCR2 |
| 1817 | 4.48 | 0 | Unmethylated | 17545522 | DCR2 |
| 1916 | 4.48 | 0 | Unmethylated | 17545522 | DCR2 |
| 2016 | 4.48 | 0 | Unmethylated | 17545522 | DCR2 |
| 2122 | 4.48 | 0 | Unmethylated | 17545522 | DCR2 |
| 2219 | 4.48 | 0 | Unmethylated | 17545522 | DCR2 |
| 2317 | 4.48 | 0 | Unmethylated | 17545522 | DCR2 |
| 2415 | 4.48 | 0 | Unmethylated | 17545522 | DCR2 |
| 2513 | 4.48 | 0 | Unmethylated | 17545522 | DCR2 |
| 2612 | 4.48 | 0 | Unmethylated | 17545522 | DCR2 |
| 2711 | 4.48 | 0 | Unmethylated | 17545522 | DCR2 |
| 2811 | 4.48 | 0 | Unmethylated | 17545522 | DCR2 |
| 2911 | 4.48 | 0 | Unmethylated | 17545522 | DCR2 |
| 3010 | 4.48 | 0 | Unmethylated | 17545522 | DCR2 |
| 3115 | 4.48 | 0 | Unmethylated | 17545522 | DCR2 |
| 3212 | 4.48 | 0 | Unmethylated | 17545522 | DCR2 |
| 249 | 4.49 | 0 | Unmethylated | 17545522 | CASP8 |
| 258 | 4.49 | 0 | Unmethylated | 17545522 | CASP8 |
| 267 | 4.49 | 0 | Unmethylated | 17545522 | CASP8 |
| 276 | 4.49 | 0 | Unmethylated | 17545522 | CASP8 |
| 286 | 4.49 | 0 | Unmethylated | 17545522 | CASP8 |
| 296 | 4.49 | 0 | Unmethylated | 17545522 | CASP8 |
| 305 | 4.49 | 0 | Unmethylated | 17545522 | CASP8 |
| 329 | 4.49 | 0 | Unmethylated | 17545522 | CASP8 |
| 428 | 4.49 | 0 | Unmethylated | 17545522 | CASP8 |
| 527 | 4.49 | 0 | Unmethylated | 17545522 | CASP8 |
| 626 | 4.49 | 0 | Unmethylated | 17545522 | CASP8 |
| 726 | 4.49 | 0 | Unmethylated | 17545522 | CASP8 |
| 826 | 4.49 | 0 | Unmethylated | 17545522 | CASP8 |
| 926 | 4.49 | 0 | Unmethylated | 17545522 | CASP8 |
| 1026 | 4.49 | 0 | Unmethylated | 17545522 | CASP8 |
| 1102 | 4.49 | 0 | Unmethylated | 17545522 | CASP8 |
| 1126 | 4.49 | 0 | Unmethylated | 17545522 | CASP8 |
| 1221 | 4.49 | 0 | Unmethylated | 17545522 | CASP8 |
| 1317 | 4.49 | 0 | Unmethylated | 17545522 | CASP8 |

| | | | | | |
|---|---|---|---|---|---|
| 1416 | 4.49 | 0 | Unmethylated | 17545522 | CASP8 |
| 1515 | 4.49 | 0 | Unmethylated | 17545522 | CASP8 |
| 1615 | 4.49 | 0 | Unmethylated | 17545522 | CASP8 |
| 1714 | 4.49 | 0 | Unmethylated | 17545522 | CASP8 |
| 1812 | 4.49 | 0 | Unmethylated | 17545522 | CASP8 |
| 1911 | 4.49 | 0 | Unmethylated | 17545522 | CASP8 |
| 2011 | 4.49 | 0 | Unmethylated | 17545522 | CASP8 |
| 2117 | 4.49 | 0 | Unmethylated | 17545522 | CASP8 |
| 2214 | 4.49 | 0 | Unmethylated | 17545522 | CASP8 |
| 2312 | 4.49 | 0 | Unmethylated | 17545522 | CASP8 |
| 2410 | 4.49 | 0 | Unmethylated | 17545522 | CASP8 |
| 3110 | 4.49 | 0 | Unmethylated | 17545522 | CASP8 |
| 919 | 4.5 | 1 | Methylated | 18980997 | DCR2 |
| 1713 | 4.6 | 1 | Methylated | 23619990 | CASP8 |
| 928 | 4.7 | 1 | Methylated | 21104989 | CASP8 |
| 1019 | 4.79 | 1 | Methylated | 18980997 | DCR2 |
| 935 | 4.86 | 1 | Unmethylated | 18980997 | DCR2 |
| 221 | 5.1 | 1 | Unmethylated | 23619990 | CASP8 |
| 1119 | 5.14 | 1 | Methylated | 18980997 | DCR2 |
| 1511 | 5.4 | 1 | Methylated | 23619990 | CASP8 |
| 1611 | 5.4 | 1 | Methylated | 23619990 | CASP8 |
| 1710 | 5.4 | 1 | Methylated | 23619990 | CASP8 |
| 1811 | 5.4 | 1 | Methylated | 23619990 | CASP8 |
| 188 | 5.6 | 1 | Methylated | 23619990 | CASP8 |
| 197 | 6.5 | 0 | Methylated | 23619990 | CASP8 |
| 207 | 6.5 | 0 | Methylated | 23619990 | CASP8 |
| 236 | 6.5 | 0 | Methylated | 23619990 | CASP8 |
| 245 | 6.5 | 0 | Methylated | 23619990 | CASP8 |
| 254 | 6.5 | 0 | Methylated | 23619990 | CASP8 |
| 263 | 6.5 | 0 | Methylated | 23619990 | CASP8 |
| 2113 | 6.5 | 0 | Methylated | 23619990 | CASP8 |
| 2210 | 6.5 | 0 | Methylated | 23619990 | CASP8 |
| 1028 | 6.51 | 1 | Methylated | 21104989 | CASP8 |
| 1128 | 6.51 | 1 | Methylated | 21104989 | CASP8 |
| 148 | 6.54 | 0 | Methylated | 17570703 | CASP8 |
| 158 | 6.54 | 0 | Methylated | 17570703 | CASP8 |
| 168 | 6.54 | 0 | Methylated | 17570703 | CASP8 |
| 177 | 6.54 | 0 | Methylated | 17570703 | CASP8 |
| 185 | 6.54 | 0 | Methylated | 17570703 | CASP8 |
| 194 | 6.54 | 0 | Methylated | 17570703 | CASP8 |
| 204 | 6.54 | 0 | Methylated | 17570703 | CASP8 |
| 224 | 6.54 | 0 | Methylated | 17570703 | CASP8 |
| 233 | 6.54 | 0 | Methylated | 17570703 | CASP8 |
| 1118 | 6.54 | 0 | Methylated | 17570703 | CASP8 |
| 1214 | 6.54 | 0 | Methylated | 17570703 | CASP8 |
| 1310 | 6.54 | 0 | Methylated | 17570703 | CASP8 |
| 2110 | 6.54 | 0 | Methylated | 17570703 | CASP8 |

| | | | | | |
|---|---|---|---|---|---|
| 346 | 6.54 | 0 | Unmethylated | 17570703 | CASP8 |
| 356 | 6.54 | 0 | Unmethylated | 17570703 | CASP8 |
| 366 | 6.54 | 0 | Unmethylated | 17570703 | CASP8 |
| 376 | 6.54 | 0 | Unmethylated | 17570703 | CASP8 |
| 386 | 6.54 | 0 | Unmethylated | 17570703 | CASP8 |
| 396 | 6.54 | 0 | Unmethylated | 17570703 | CASP8 |
| 1033 | 6.54 | 0 | Unmethylated | 17570703 | CASP8 |
| 1133 | 6.54 | 0 | Unmethylated | 17570703 | CASP8 |
| 1227 | 6.54 | 0 | Unmethylated | 17570703 | CASP8 |
| 1323 | 6.54 | 0 | Unmethylated | 17570703 | CASP8 |
| 1422 | 6.54 | 0 | Unmethylated | 17570703 | CASP8 |
| 1521 | 6.54 | 0 | Unmethylated | 17570703 | CASP8 |
| 1621 | 6.54 | 0 | Unmethylated | 17570703 | CASP8 |
| 1720 | 6.54 | 0 | Unmethylated | 17570703 | CASP8 |
| 1818 | 6.54 | 0 | Unmethylated | 17570703 | CASP8 |
| 1917 | 6.54 | 0 | Unmethylated | 17570703 | CASP8 |
| 2017 | 6.54 | 0 | Unmethylated | 17570703 | CASP8 |
| 2123 | 6.54 | 0 | Unmethylated | 17570703 | CASP8 |
| 2220 | 6.54 | 0 | Unmethylated | 17570703 | CASP8 |
| 2318 | 6.54 | 0 | Unmethylated | 17570703 | CASP8 |
| 2416 | 6.54 | 0 | Unmethylated | 17570703 | CASP8 |
| 2514 | 6.54 | 0 | Unmethylated | 17570703 | CASP8 |
| 2613 | 6.54 | 0 | Unmethylated | 17570703 | CASP8 |
| 2712 | 6.54 | 0 | Unmethylated | 17570703 | CASP8 |
| 2812 | 6.54 | 0 | Unmethylated | 17570703 | CASP8 |
| 2912 | 6.54 | 0 | Unmethylated | 17570703 | CASP8 |
| 3011 | 6.54 | 0 | Unmethylated | 17570703 | CASP8 |
| 3116 | 6.54 | 0 | Unmethylated | 17570703 | CASP8 |
| 3213 | 6.54 | 0 | Unmethylated | 17570703 | CASP8 |
| 3310 | 6.54 | 0 | Unmethylated | 17570703 | CASP8 |
| 1717 | 7.27 | 1 | Methylated | 15623630 | RASSF1 |
| 1815 | 7.74 | 1 | Methylated | 15623630 | RASSF1 |
| 23 | 7.8 | 0 | Unmethylated | 23619990 | CASP8 |
| 24 | 7.8 | 0 | Unmethylated | 23619990 | CASP8 |
| 25 | 7.8 | 0 | Unmethylated | 23619990 | CASP8 |
| 26 | 7.8 | 0 | Unmethylated | 23619990 | CASP8 |
| 27 | 7.8 | 0 | Unmethylated | 23619990 | CASP8 |
| 28 | 7.8 | 0 | Unmethylated | 23619990 | CASP8 |
| 29 | 7.8 | 0 | Unmethylated | 23619990 | CASP8 |
| 30 | 7.8 | 0 | Unmethylated | 23619990 | CASP8 |
| 31 | 7.8 | 0 | Unmethylated | 23619990 | CASP8 |
| 32 | 7.8 | 0 | Unmethylated | 23619990 | CASP8 |
| 33 | 7.8 | 0 | Unmethylated | 23619990 | CASP8 |
| 34 | 7.8 | 0 | Unmethylated | 23619990 | CASP8 |
| 35 | 7.8 | 0 | Unmethylated | 23619990 | CASP8 |
| 36 | 7.8 | 0 | Unmethylated | 23619990 | CASP8 |
| 37 | 7.8 | 0 | Unmethylated | 23619990 | CASP8 |

| | | | | | |
|---|---|---|---|---|---|
| 38 | 7.8 | 0 | Unmethylated | 23619990 | CASP8 |
| 39 | 7.8 | 0 | Unmethylated | 23619990 | CASP8 |
| 40 | 7.8 | 0 | Unmethylated | 23619990 | CASP8 |
| 41 | 7.8 | 0 | Unmethylated | 23619990 | CASP8 |
| 42 | 7.8 | 0 | Unmethylated | 23619990 | CASP8 |
| 43 | 7.8 | 0 | Unmethylated | 23619990 | CASP8 |
| 44 | 7.8 | 0 | Unmethylated | 23619990 | CASP8 |
| 45 | 7.8 | 0 | Unmethylated | 23619990 | CASP8 |
| 46 | 7.8 | 0 | Unmethylated | 23619990 | CASP8 |
| 47 | 7.8 | 0 | Unmethylated | 23619990 | CASP8 |
| 48 | 7.8 | 0 | Unmethylated | 23619990 | CASP8 |
| 49 | 7.8 | 0 | Unmethylated | 23619990 | CASP8 |
| 50 | 7.8 | 0 | Unmethylated | 23619990 | CASP8 |
| 51 | 7.8 | 0 | Unmethylated | 23619990 | CASP8 |
| 52 | 7.8 | 0 | Unmethylated | 23619990 | CASP8 |
| 53 | 7.8 | 0 | Unmethylated | 23619990 | CASP8 |
| 54 | 7.8 | 0 | Unmethylated | 23619990 | CASP8 |
| 55 | 7.8 | 0 | Unmethylated | 23619990 | CASP8 |
| 56 | 7.8 | 0 | Unmethylated | 23619990 | CASP8 |
| 57 | 7.8 | 0 | Unmethylated | 23619990 | CASP8 |
| 58 | 7.8 | 0 | Unmethylated | 23619990 | CASP8 |
| 59 | 7.8 | 0 | Unmethylated | 23619990 | CASP8 |
| 60 | 7.8 | 0 | Unmethylated | 23619990 | CASP8 |
| 61 | 7.8 | 0 | Unmethylated | 23619990 | CASP8 |
| 62 | 7.8 | 0 | Unmethylated | 23619990 | CASP8 |
| 63 | 7.8 | 0 | Unmethylated | 23619990 | CASP8 |
| 64 | 7.8 | 0 | Unmethylated | 23619990 | CASP8 |
| 65 | 7.8 | 0 | Unmethylated | 23619990 | CASP8 |
| 66 | 7.8 | 0 | Unmethylated | 23619990 | CASP8 |
| 67 | 7.8 | 0 | Unmethylated | 23619990 | CASP8 |
| 68 | 7.8 | 0 | Unmethylated | 23619990 | CASP8 |
| 69 | 7.8 | 0 | Unmethylated | 23619990 | CASP8 |
| 70 | 7.8 | 0 | Unmethylated | 23619990 | CASP8 |
| 71 | 7.8 | 0 | Unmethylated | 23619990 | CASP8 |
| 72 | 7.8 | 0 | Unmethylated | 23619990 | CASP8 |
| 73 | 7.8 | 0 | Unmethylated | 23619990 | CASP8 |
| 74 | 7.8 | 0 | Unmethylated | 23619990 | CASP8 |
| 75 | 7.8 | 0 | Unmethylated | 23619990 | CASP8 |
| 76 | 7.8 | 0 | Unmethylated | 23619990 | CASP8 |
| 77 | 7.8 | 0 | Unmethylated | 23619990 | CASP8 |
| 78 | 7.8 | 0 | Unmethylated | 23619990 | CASP8 |
| 79 | 7.8 | 0 | Unmethylated | 23619990 | CASP8 |
| 80 | 7.8 | 0 | Unmethylated | 23619990 | CASP8 |
| 81 | 7.8 | 0 | Unmethylated | 23619990 | CASP8 |
| 82 | 7.8 | 0 | Unmethylated | 23619990 | CASP8 |
| 83 | 7.8 | 0 | Unmethylated | 23619990 | CASP8 |
| 84 | 7.8 | 0 | Unmethylated | 23619990 | CASP8 |

| | | | | | |
|---|---|---|---|---|---|
| 85 | 7.8 | 0 | Unmethylated | 23619990 | CASP8 |
| 86 | 7.8 | 0 | Unmethylated | 23619990 | CASP8 |
| 87 | 7.8 | 0 | Unmethylated | 23619990 | CASP8 |
| 88 | 7.8 | 0 | Unmethylated | 23619990 | CASP8 |
| 89 | 7.8 | 0 | Unmethylated | 23619990 | CASP8 |
| 90 | 7.8 | 0 | Unmethylated | 23619990 | CASP8 |
| 91 | 7.8 | 0 | Unmethylated | 23619990 | CASP8 |
| 92 | 7.8 | 0 | Unmethylated | 23619990 | CASP8 |
| 93 | 7.8 | 0 | Unmethylated | 23619990 | CASP8 |
| 94 | 7.8 | 0 | Unmethylated | 23619990 | CASP8 |
| 95 | 7.8 | 0 | Unmethylated | 23619990 | CASP8 |
| 96 | 7.8 | 0 | Unmethylated | 23619990 | CASP8 |
| 97 | 7.8 | 0 | Unmethylated | 23619990 | CASP8 |
| 98 | 7.8 | 0 | Unmethylated | 23619990 | CASP8 |
| 99 | 7.8 | 0 | Unmethylated | 23619990 | CASP8 |
| 100 | 7.8 | 0 | Unmethylated | 23619990 | CASP8 |
| 101 | 7.8 | 0 | Unmethylated | 23619990 | CASP8 |
| 102 | 7.8 | 0 | Unmethylated | 23619990 | CASP8 |
| 103 | 7.8 | 0 | Unmethylated | 23619990 | CASP8 |
| 104 | 7.8 | 0 | Unmethylated | 23619990 | CASP8 |
| 105 | 7.8 | 0 | Unmethylated | 23619990 | CASP8 |
| 106 | 7.8 | 0 | Unmethylated | 23619990 | CASP8 |
| 107 | 7.8 | 0 | Unmethylated | 23619990 | CASP8 |
| 108 | 7.8 | 0 | Unmethylated | 23619990 | CASP8 |
| 109 | 7.8 | 0 | Unmethylated | 23619990 | CASP8 |
| 110 | 7.8 | 0 | Unmethylated | 23619990 | CASP8 |
| 111 | 7.8 | 0 | Unmethylated | 23619990 | CASP8 |
| 112 | 7.8 | 0 | Unmethylated | 23619990 | CASP8 |
| 113 | 7.8 | 0 | Unmethylated | 23619990 | CASP8 |
| 114 | 7.8 | 0 | Unmethylated | 23619990 | CASP8 |
| 348 | 7.88 | 0 | Methylated | 17570703 | RASSF1 |
| 357 | 7.88 | 0 | Methylated | 17570703 | RASSF1 |
| 367 | 7.88 | 0 | Methylated | 17570703 | RASSF1 |
| 377 | 7.88 | 0 | Methylated | 17570703 | RASSF1 |
| 387 | 7.88 | 0 | Methylated | 17570703 | RASSF1 |
| 397 | 7.88 | 0 | Methylated | 17570703 | RASSF1 |
| 403 | 7.88 | 0 | Methylated | 17570703 | RASSF1 |
| 438 | 7.88 | 0 | Methylated | 17570703 | RASSF1 |
| 443 | 7.88 | 0 | Methylated | 17570703 | RASSF1 |
| 453 | 7.88 | 0 | Methylated | 17570703 | RASSF1 |
| 463 | 7.88 | 0 | Methylated | 17570703 | RASSF1 |
| 473 | 7.88 | 0 | Methylated | 17570703 | RASSF1 |
| 483 | 7.88 | 0 | Methylated | 17570703 | RASSF1 |
| 493 | 7.88 | 0 | Methylated | 17570703 | RASSF1 |
| 503 | 7.88 | 0 | Methylated | 17570703 | RASSF1 |
| 536 | 7.88 | 0 | Methylated | 17570703 | RASSF1 |
| 542 | 7.88 | 0 | Methylated | 17570703 | RASSF1 |

| | | | | | |
|---|---|---|---|---|---|
| 552 | 7.88 | 0 | Methylated | 17570703 | RASSF1 |
| 562 | 7.88 | 0 | Methylated | 17570703 | RASSF1 |
| 572 | 7.88 | 0 | Methylated | 17570703 | RASSF1 |
| 582 | 7.88 | 0 | Methylated | 17570703 | RASSF1 |
| 592 | 7.88 | 0 | Methylated | 17570703 | RASSF1 |
| 2018 | 7.88 | 0 | Methylated | 17570703 | RASSF1 |
| 2124 | 7.88 | 0 | Methylated | 17570703 | RASSF1 |
| 2221 | 7.88 | 0 | Methylated | 17570703 | RASSF1 |
| 2319 | 7.88 | 0 | Methylated | 17570703 | RASSF1 |
| 2417 | 7.88 | 0 | Methylated | 17570703 | RASSF1 |
| 2515 | 7.88 | 0 | Methylated | 17570703 | RASSF1 |
| 2614 | 7.88 | 0 | Methylated | 17570703 | RASSF1 |
| 2713 | 7.88 | 0 | Methylated | 17570703 | RASSF1 |
| 2813 | 7.88 | 0 | Methylated | 17570703 | RASSF1 |
| 2913 | 7.88 | 0 | Methylated | 17570703 | RASSF1 |
| 3012 | 7.88 | 0 | Methylated | 17570703 | RASSF1 |
| 3117 | 7.88 | 0 | Methylated | 17570703 | RASSF1 |
| 3214 | 7.88 | 0 | Methylated | 17570703 | RASSF1 |
| 3311 | 7.88 | 0 | Methylated | 17570703 | RASSF1 |
| 4110 | 7.88 | 0 | Methylated | 17570703 | RASSF1 |
| 4210 | 7.88 | 0 | Methylated | 17570703 | RASSF1 |
| 5110 | 7.88 | 0 | Methylated | 17570703 | RASSF1 |
| 5210 | 7.88 | 0 | Methylated | 17570703 | RASSF1 |
| 180 | 7.88 | 0 | Unmethylated | 17570703 | RASSF1 |
| 238 | 7.88 | 0 | Unmethylated | 17570703 | RASSF1 |
| 326 | 7.88 | 0 | Unmethylated | 17570703 | RASSF1 |
| 1914 | 8.5 | 1 | Methylated | 15623630 | RASSF1 |
| 2014 | 8.5 | 1 | Methylated | 15623630 | RASSF1 |
| 115 | 8.5 | 0 | Unmethylated | 23619990 | CASP8 |
| 116 | 8.5 | 0 | Unmethylated | 23619990 | CASP8 |
| 117 | 8.5 | 0 | Unmethylated | 23619990 | CASP8 |
| 118 | 8.5 | 0 | Unmethylated | 23619990 | CASP8 |
| 119 | 8.5 | 0 | Unmethylated | 23619990 | CASP8 |
| 120 | 8.5 | 0 | Unmethylated | 23619990 | CASP8 |
| 121 | 8.5 | 0 | Unmethylated | 23619990 | CASP8 |
| 122 | 8.5 | 0 | Unmethylated | 23619990 | CASP8 |
| 231 | 8.5 | 0 | Unmethylated | 23619990 | CASP8 |
| 241 | 8.5 | 0 | Unmethylated | 23619990 | CASP8 |
| 251 | 8.5 | 0 | Unmethylated | 23619990 | CASP8 |
| 261 | 8.5 | 0 | Unmethylated | 23619990 | CASP8 |
| 271 | 8.5 | 0 | Unmethylated | 23619990 | CASP8 |
| 281 | 8.5 | 0 | Unmethylated | 23619990 | CASP8 |
| 291 | 8.5 | 0 | Unmethylated | 23619990 | CASP8 |
| 301 | 8.5 | 0 | Unmethylated | 23619990 | CASP8 |
| 311 | 8.5 | 0 | Unmethylated | 23619990 | CASP8 |
| 321 | 8.5 | 0 | Unmethylated | 23619990 | CASP8 |
| 331 | 8.5 | 0 | Unmethylated | 23619990 | CASP8 |

| | | | | | |
|---|---|---|---|---|---|
| 341 | 8.5 | 0 | Unmethylated | 23619990 | CASP8 |
| 351 | 8.5 | 0 | Unmethylated | 23619990 | CASP8 |
| 361 | 8.5 | 0 | Unmethylated | 23619990 | CASP8 |
| 371 | 8.5 | 0 | Unmethylated | 23619990 | CASP8 |
| 381 | 8.5 | 0 | Unmethylated | 23619990 | CASP8 |
| 391 | 8.5 | 0 | Unmethylated | 23619990 | CASP8 |
| 401 | 8.5 | 0 | Unmethylated | 23619990 | CASP8 |
| 411 | 8.5 | 0 | Unmethylated | 23619990 | CASP8 |
| 421 | 8.5 | 0 | Unmethylated | 23619990 | CASP8 |
| 431 | 8.5 | 0 | Unmethylated | 23619990 | CASP8 |
| 441 | 8.5 | 0 | Unmethylated | 23619990 | CASP8 |
| 451 | 8.5 | 0 | Unmethylated | 23619990 | CASP8 |
| 461 | 8.5 | 0 | Unmethylated | 23619990 | CASP8 |
| 471 | 8.5 | 0 | Unmethylated | 23619990 | CASP8 |
| 481 | 8.5 | 0 | Unmethylated | 23619990 | CASP8 |
| 491 | 8.5 | 0 | Unmethylated | 23619990 | CASP8 |
| 501 | 8.5 | 0 | Unmethylated | 23619990 | CASP8 |
| 511 | 8.5 | 0 | Unmethylated | 23619990 | CASP8 |
| 521 | 8.5 | 0 | Unmethylated | 23619990 | CASP8 |
| 531 | 8.5 | 0 | Unmethylated | 23619990 | CASP8 |
| 541 | 8.5 | 0 | Unmethylated | 23619990 | CASP8 |
| 551 | 8.5 | 0 | Unmethylated | 23619990 | CASP8 |
| 561 | 8.5 | 0 | Unmethylated | 23619990 | CASP8 |
| 571 | 8.5 | 0 | Unmethylated | 23619990 | CASP8 |
| 581 | 8.5 | 0 | Unmethylated | 23619990 | CASP8 |
| 591 | 8.5 | 0 | Unmethylated | 23619990 | CASP8 |
| 601 | 8.5 | 0 | Unmethylated | 23619990 | CASP8 |
| 611 | 8.5 | 0 | Unmethylated | 23619990 | CASP8 |
| 621 | 8.5 | 0 | Unmethylated | 23619990 | CASP8 |
| 631 | 8.5 | 0 | Unmethylated | 23619990 | CASP8 |
| 641 | 8.5 | 0 | Unmethylated | 23619990 | CASP8 |
| 651 | 8.5 | 0 | Unmethylated | 23619990 | CASP8 |
| 661 | 8.5 | 0 | Unmethylated | 23619990 | CASP8 |
| 671 | 8.5 | 0 | Unmethylated | 23619990 | CASP8 |
| 681 | 8.5 | 0 | Unmethylated | 23619990 | CASP8 |
| 691 | 8.5 | 0 | Unmethylated | 23619990 | CASP8 |
| 701 | 8.5 | 0 | Unmethylated | 23619990 | CASP8 |
| 711 | 8.5 | 0 | Unmethylated | 23619990 | CASP8 |
| 721 | 8.5 | 0 | Unmethylated | 23619990 | CASP8 |
| 731 | 8.5 | 0 | Unmethylated | 23619990 | CASP8 |
| 741 | 8.5 | 0 | Unmethylated | 23619990 | CASP8 |
| 751 | 8.5 | 0 | Unmethylated | 23619990 | CASP8 |
| 761 | 8.5 | 0 | Unmethylated | 23619990 | CASP8 |
| 771 | 8.5 | 0 | Unmethylated | 23619990 | CASP8 |
| 781 | 8.5 | 0 | Unmethylated | 23619990 | CASP8 |
| 791 | 8.5 | 0 | Unmethylated | 23619990 | CASP8 |
| 801 | 8.5 | 0 | Unmethylated | 23619990 | CASP8 |

| | | | | | |
|---|---|---|---|---|---|
| 811 | 8.5 | 0 | Unmethylated | 23619990 | CASP8 |
| 821 | 8.5 | 0 | Unmethylated | 23619990 | CASP8 |
| 831 | 8.5 | 0 | Unmethylated | 23619990 | CASP8 |
| 841 | 8.5 | 0 | Unmethylated | 23619990 | CASP8 |
| 851 | 8.5 | 0 | Unmethylated | 23619990 | CASP8 |
| 861 | 8.5 | 0 | Unmethylated | 23619990 | CASP8 |
| 871 | 8.5 | 0 | Unmethylated | 23619990 | CASP8 |
| 881 | 8.5 | 0 | Unmethylated | 23619990 | CASP8 |
| 891 | 8.5 | 0 | Unmethylated | 23619990 | CASP8 |
| 901 | 8.5 | 0 | Unmethylated | 23619990 | CASP8 |
| 911 | 8.5 | 0 | Unmethylated | 23619990 | CASP8 |
| 921 | 8.5 | 0 | Unmethylated | 23619990 | CASP8 |
| 931 | 8.5 | 0 | Unmethylated | 23619990 | CASP8 |
| 941 | 8.5 | 0 | Unmethylated | 23619990 | CASP8 |
| 951 | 8.5 | 0 | Unmethylated | 23619990 | CASP8 |
| 961 | 8.5 | 0 | Unmethylated | 23619990 | CASP8 |
| 971 | 8.5 | 0 | Unmethylated | 23619990 | CASP8 |
| 981 | 8.5 | 0 | Unmethylated | 23619990 | CASP8 |
| 991 | 8.5 | 0 | Unmethylated | 23619990 | CASP8 |
| 1001 | 8.5 | 0 | Unmethylated | 23619990 | CASP8 |
| 1011 | 8.5 | 0 | Unmethylated | 23619990 | CASP8 |
| 1021 | 8.5 | 0 | Unmethylated | 23619990 | CASP8 |
| 1031 | 8.5 | 0 | Unmethylated | 23619990 | CASP8 |
| 1041 | 8.5 | 0 | Unmethylated | 23619990 | CASP8 |
| 1051 | 8.5 | 0 | Unmethylated | 23619990 | CASP8 |
| 1061 | 8.5 | 0 | Unmethylated | 23619990 | CASP8 |
| 1071 | 8.5 | 0 | Unmethylated | 23619990 | CASP8 |
| 1081 | 8.5 | 0 | Unmethylated | 23619990 | CASP8 |
| 1091 | 8.5 | 0 | Unmethylated | 23619990 | CASP8 |
| 1101 | 8.5 | 0 | Unmethylated | 23619990 | CASP8 |
| 1111 | 8.5 | 0 | Unmethylated | 23619990 | CASP8 |
| 1121 | 8.5 | 0 | Unmethylated | 23619990 | CASP8 |
| 1131 | 8.5 | 0 | Unmethylated | 23619990 | CASP8 |
| 1141 | 8.5 | 0 | Unmethylated | 23619990 | CASP8 |
| 248 | 11 | 1 | Methylated | 23619990 | CASP8 |
| 257 | 11 | 1 | Methylated | 23619990 | CASP8 |
| 266 | 11 | 1 | Methylated | 23619990 | CASP8 |
| 275 | 11 | 1 | Methylated | 23619990 | CASP8 |
| 285 | 11 | 1 | Methylated | 23619990 | CASP8 |
| 295 | 11 | 1 | Methylated | 23619990 | CASP8 |
| 304 | 11 | 0 | Methylated | 23619990 | CASP8 |
| 1910 | 11 | 1 | Methylated | 23619990 | CASP8 |
| 2010 | 11 | 1 | Methylated | 23619990 | CASP8 |
| 2116 | 11 | 1 | Methylated | 23619990 | CASP8 |
| 2213 | 11 | 1 | Methylated | 23619990 | CASP8 |
| 2311 | 11 | 1 | Methylated | 23619990 | CASP8 |
| 269 | 11.1 | 0 | Methylated | 21104989 | CASP8 |

| | | | | | |
|---|---|---|---|---|---|
| 278 | 11.1 | 0 | Methylated | 21104989 | CASP8 |
| 288 | 11.1 | 0 | Methylated | 21104989 | CASP8 |
| 298 | 11.1 | 0 | Methylated | 21104989 | CASP8 |
| 307 | 11.1 | 0 | Methylated | 21104989 | CASP8 |
| 1223 | 11.1 | 0 | Methylated | 21104989 | CASP8 |
| 1319 | 11.1 | 0 | Methylated | 21104989 | CASP8 |
| 1418 | 11.1 | 0 | Methylated | 21104989 | CASP8 |
| 1517 | 11.1 | 0 | Methylated | 21104989 | CASP8 |
| 1617 | 11.1 | 0 | Methylated | 21104989 | CASP8 |
| 1716 | 11.1 | 0 | Methylated | 21104989 | CASP8 |
| 1814 | 11.1 | 0 | Methylated | 21104989 | CASP8 |
| 1913 | 11.1 | 0 | Methylated | 21104989 | CASP8 |
| 2013 | 11.1 | 0 | Methylated | 21104989 | CASP8 |
| 2119 | 11.1 | 0 | Methylated | 21104989 | CASP8 |
| 2216 | 11.1 | 0 | Methylated | 21104989 | CASP8 |
| 2314 | 11.1 | 0 | Methylated | 21104989 | CASP8 |
| 2412 | 11.1 | 0 | Methylated | 21104989 | CASP8 |
| 2510 | 11.1 | 0 | Methylated | 21104989 | CASP8 |
| 3112 | 11.1 | 0 | Methylated | 21104989 | CASP8 |
| 128 | 13.5 | 0 | Unmethylated | 15623630 | RASSF1 |
| 133 | 13.5 | 0 | Unmethylated | 15623630 | RASSF1 |
| 143 | 13.5 | 0 | Unmethylated | 15623630 | RASSF1 |
| 153 | 13.5 | 0 | Unmethylated | 15623630 | RASSF1 |
| 163 | 13.5 | 0 | Unmethylated | 15623630 | RASSF1 |
| 172 | 13.5 | 0 | Unmethylated | 15623630 | RASSF1 |
| 213 | 13.5 | 0 | Unmethylated | 15623630 | RASSF1 |
| 313 | 13.5 | 0 | Unmethylated | 15623630 | RASSF1 |
| 413 | 13.5 | 0 | Unmethylated | 15623630 | RASSF1 |
| 513 | 13.5 | 0 | Unmethylated | 15623630 | RASSF1 |
| 613 | 13.5 | 0 | Unmethylated | 15623630 | RASSF1 |
| 713 | 13.5 | 0 | Unmethylated | 15623630 | RASSF1 |
| 813 | 13.5 | 0 | Unmethylated | 15623630 | RASSF1 |
| 913 | 13.5 | 0 | Unmethylated | 15623630 | RASSF1 |
| 1013 | 13.5 | 0 | Unmethylated | 15623630 | RASSF1 |
| 1113 | 13.5 | 0 | Unmethylated | 15623630 | RASSF1 |
| 189 | 14.8 | 0 | Unmethylated | 21104989 | CASP8 |
| 198 | 14.8 | 0 | Unmethylated | 21104989 | CASP8 |
| 208 | 14.8 | 0 | Unmethylated | 21104989 | CASP8 |
| 230 | 14.8 | 0 | Unmethylated | 21104989 | CASP8 |
| 237 | 14.8 | 0 | Unmethylated | 21104989 | CASP8 |
| 246 | 14.8 | 0 | Unmethylated | 21104989 | CASP8 |
| 255 | 14.8 | 0 | Unmethylated | 21104989 | CASP8 |
| 264 | 14.8 | 0 | Unmethylated | 21104989 | CASP8 |
| 273 | 14.8 | 0 | Unmethylated | 21104989 | CASP8 |
| 283 | 14.8 | 0 | Unmethylated | 21104989 | CASP8 |
| 293 | 14.8 | 0 | Unmethylated | 21104989 | CASP8 |
| 325 | 14.8 | 0 | Unmethylated | 21104989 | CASP8 |

| | | | | | |
|---|---|---|---|---|---|
| 425 | 14.8 | 0 | Unmethylated | 21104989 | CASP8 |
| 524 | 14.8 | 0 | Unmethylated | 21104989 | CASP8 |
| 623 | 14.8 | 0 | Unmethylated | 21104989 | CASP8 |
| 723 | 14.8 | 0 | Unmethylated | 21104989 | CASP8 |
| 823 | 14.8 | 0 | Unmethylated | 21104989 | CASP8 |
| 923 | 14.8 | 0 | Unmethylated | 21104989 | CASP8 |
| 1023 | 14.8 | 0 | Unmethylated | 21104989 | CASP8 |
| 1123 | 14.8 | 0 | Unmethylated | 21104989 | CASP8 |
| 1218 | 14.8 | 0 | Unmethylated | 21104989 | CASP8 |
| 1314 | 14.8 | 0 | Unmethylated | 21104989 | CASP8 |
| 1413 | 14.8 | 0 | Unmethylated | 21104989 | CASP8 |
| 1512 | 14.8 | 0 | Unmethylated | 21104989 | CASP8 |
| 1612 | 14.8 | 0 | Unmethylated | 21104989 | CASP8 |
| 1711 | 14.8 | 0 | Unmethylated | 21104989 | CASP8 |
| 2114 | 14.8 | 0 | Unmethylated | 21104989 | CASP8 |
| 2211 | 14.8 | 0 | Unmethylated | 21104989 | CASP8 |
| 279 | 16.1 | 0 | Methylated | 15623630 | RASSF1 |
| 289 | 16.1 | 0 | Methylated | 15623630 | RASSF1 |
| 299 | 16.1 | 0 | Methylated | 15623630 | RASSF1 |
| 308 | 16.1 | 0 | Methylated | 15623630 | RASSF1 |
| 335 | 16.1 | 0 | Methylated | 15623630 | RASSF1 |
| 343 | 16.1 | 0 | Methylated | 15623630 | RASSF1 |
| 353 | 16.1 | 0 | Methylated | 15623630 | RASSF1 |
| 363 | 16.1 | 0 | Methylated | 15623630 | RASSF1 |
| 373 | 16.1 | 0 | Methylated | 15623630 | RASSF1 |
| 383 | 16.1 | 0 | Methylated | 15623630 | RASSF1 |
| 393 | 16.1 | 0 | Methylated | 15623630 | RASSF1 |
| 2120 | 16.1 | 0 | Methylated | 15623630 | RASSF1 |
| 2217 | 16.1 | 0 | Methylated | 15623630 | RASSF1 |
| 2315 | 16.1 | 0 | Methylated | 15623630 | RASSF1 |
| 2413 | 16.1 | 0 | Methylated | 15623630 | RASSF1 |
| 2511 | 16.1 | 0 | Methylated | 15623630 | RASSF1 |
| 2610 | 16.1 | 0 | Methylated | 15623630 | RASSF1 |
| 3113 | 16.1 | 0 | Methylated | 15623630 | RASSF1 |
| 3210 | 16.1 | 0 | Methylated | 15623630 | RASSF1 |
| 159 | 16.9 | 0 | Methylated | 18980997 | DCR2 |
| 169 | 16.9 | 0 | Methylated | 18980997 | DCR2 |
| 178 | 16.9 | 0 | Methylated | 18980997 | DCR2 |
| 186 | 16.9 | 0 | Methylated | 18980997 | DCR2 |
| 195 | 16.9 | 0 | Methylated | 18980997 | DCR2 |
| 205 | 16.9 | 0 | Methylated | 18980997 | DCR2 |
| 226 | 16.9 | 0 | Methylated | 18980997 | DCR2 |
| 234 | 16.9 | 0 | Methylated | 18980997 | DCR2 |
| 243 | 16.9 | 0 | Methylated | 18980997 | DCR2 |
| 1215 | 16.9 | 0 | Methylated | 18980997 | DCR2 |
| 1311 | 16.9 | 0 | Methylated | 18980997 | DCR2 |
| 1410 | 16.9 | 0 | Methylated | 18980997 | DCR2 |

| | | | | | |
|---|---|---|---|---|---|
| 2111 | 16.9 | 0 | Methylated | 18980997 | DCR2 |
| 358 | 22.9 | 0 | Unmethylated | 18980997 | DCR2 |
| 368 | 22.9 | 0 | Unmethylated | 18980997 | DCR2 |
| 378 | 22.9 | 0 | Unmethylated | 18980997 | DCR2 |
| 388 | 22.9 | 0 | Unmethylated | 18980997 | DCR2 |
| 398 | 22.9 | 0 | Unmethylated | 18980997 | DCR2 |
| 404 | 22.9 | 0 | Unmethylated | 18980997 | DCR2 |
| 444 | 22.9 | 0 | Unmethylated | 18980997 | DCR2 |
| 454 | 22.9 | 0 | Unmethylated | 18980997 | DCR2 |
| 464 | 22.9 | 0 | Unmethylated | 18980997 | DCR2 |
| 474 | 22.9 | 0 | Unmethylated | 18980997 | DCR2 |
| 484 | 22.9 | 0 | Unmethylated | 18980997 | DCR2 |
| 494 | 22.9 | 0 | Unmethylated | 18980997 | DCR2 |
| 504 | 22.9 | 0 | Unmethylated | 18980997 | DCR2 |
| 538 | 22.9 | 0 | Unmethylated | 18980997 | DCR2 |
| 543 | 22.9 | 0 | Unmethylated | 18980997 | DCR2 |
| 553 | 22.9 | 0 | Unmethylated | 18980997 | DCR2 |
| 563 | 22.9 | 0 | Unmethylated | 18980997 | DCR2 |
| 573 | 22.9 | 0 | Unmethylated | 18980997 | DCR2 |
| 583 | 22.9 | 0 | Unmethylated | 18980997 | DCR2 |
| 593 | 22.9 | 0 | Unmethylated | 18980997 | DCR2 |
| 602 | 22.9 | 0 | Unmethylated | 18980997 | DCR2 |
| 1035 | 22.9 | 0 | Unmethylated | 18980997 | DCR2 |
| 1136 | 22.9 | 0 | Unmethylated | 18980997 | DCR2 |
| 1229 | 22.9 | 0 | Unmethylated | 18980997 | DCR2 |
| 1325 | 22.9 | 0 | Unmethylated | 18980997 | DCR2 |
| 1424 | 22.9 | 0 | Unmethylated | 18980997 | DCR2 |
| 1523 | 22.9 | 0 | Unmethylated | 18980997 | DCR2 |
| 1623 | 22.9 | 0 | Unmethylated | 18980997 | DCR2 |
| 1722 | 22.9 | 0 | Unmethylated | 18980997 | DCR2 |
| 1820 | 22.9 | 0 | Unmethylated | 18980997 | DCR2 |
| 1919 | 22.9 | 0 | Unmethylated | 18980997 | DCR2 |
| 2019 | 22.9 | 0 | Unmethylated | 18980997 | DCR2 |
| 2125 | 22.9 | 0 | Unmethylated | 18980997 | DCR2 |
| 2222 | 22.9 | 0 | Unmethylated | 18980997 | DCR2 |
| 2320 | 22.9 | 0 | Unmethylated | 18980997 | DCR2 |
| 2418 | 22.9 | 0 | Unmethylated | 18980997 | DCR2 |
| 2516 | 22.9 | 0 | Unmethylated | 18980997 | DCR2 |
| 2615 | 22.9 | 0 | Unmethylated | 18980997 | DCR2 |
| 2714 | 22.9 | 0 | Unmethylated | 18980997 | DCR2 |
| 2814 | 22.9 | 0 | Unmethylated | 18980997 | DCR2 |
| 2914 | 22.9 | 0 | Unmethylated | 18980997 | DCR2 |
| 3013 | 22.9 | 0 | Unmethylated | 18980997 | DCR2 |
| 3118 | 22.9 | 0 | Unmethylated | 18980997 | DCR2 |
| 3215 | 22.9 | 0 | Unmethylated | 18980997 | DCR2 |
| 3312 | 22.9 | 0 | Unmethylated | 18980997 | DCR2 |
| 3410 | 22.9 | 0 | Unmethylated | 18980997 | DCR2 |

| | | | | | |
|---|---|---|---|---|---|
| 4111 | 22.9 | 0 | Unmethylated | 18980997 | DCR2 |
| 4211 | 22.9 | 0 | Unmethylated | 18980997 | DCR2 |
| 4310 | 22.9 | 0 | Unmethylated | 18980997 | DCR2 |
| 5111 | 22.9 | 0 | Unmethylated | 18980997 | DCR2 |
| 5211 | 22.9 | 0 | Unmethylated | 18980997 | DCR2 |
| 6110 | 22.9 | 0 | Unmethylated | 18980997 | DCR2 |
| 6210 | 22.9 | 0 | Unmethylated | 18980997 | DCR2 |
| 10000 | 0 | 1 | Unmethylated | 24680815 | CASP8 |
| 10001 | 0 | 1 | Unmethylated | 24680815 | CASP8 |
| 10002 | 0.1208 | 1 | Unmethylated | 24680815 | CASP8 |
| 10003 | 0.2425 | 1 | Unmethylated | 24680815 | CASP8 |
| 10004 | 0.7025 | 1 | Unmethylated | 24680815 | CASP8 |
| 10005 | 0.8233 | 1 | Unmethylated | 24680815 | CASP8 |
| 10006 | 0.8233 | 1 | Unmethylated | 24680815 | CASP8 |
| 10007 | 0.8233 | 1 | Unmethylated | 24680815 | CASP8 |
| 10008 | 1.3833 | 1 | Unmethylated | 24680815 | CASP8 |
| 10009 | 1.6 | 1 | Unmethylated | 24680815 | CASP8 |
| 10010 | 1.6 | 1 | Unmethylated | 24680815 | CASP8 |
| 10011 | 3.075 | 1 | Unmethylated | 24680815 | CASP8 |
| 10012 | 3.075 | 1 | Unmethylated | 24680815 | CASP8 |
| 10013 | 3.5167 | 1 | Unmethylated | 24680815 | CASP8 |
| 10014 | 3.5167 | 1 | Unmethylated | 24680815 | CASP8 |
| 10015 | 3.7083 | 1 | Unmethylated | 24680815 | CASP8 |
| 10016 | 4.2917 | 1 | Unmethylated | 24680815 | CASP8 |
| 10017 | 4.2917 | 1 | Unmethylated | 24680815 | CASP8 |
| 10018 | 5.1333 | 1 | Unmethylated | 24680815 | CASP8 |
| 10019 | 5.1333 | 1 | Unmethylated | 24680815 | CASP8 |
| 10020 | 6.5667 | 1 | Unmethylated | 24680815 | CASP8 |
| 10021 | 6.5667 | 1 | Unmethylated | 24680815 | CASP8 |
| 10022 | 6.5667 | 1 | Unmethylated | 24680815 | CASP8 |
| 10023 | 16.0833 | 0 | Unmethylated | 24680815 | CASP8 |
| 10024 | 16.0833 | 0 | Unmethylated | 24680815 | CASP8 |
| 10025 | 16.0833 | 0 | Unmethylated | 24680815 | CASP8 |
| 10026 | 16.0833 | 0 | Unmethylated | 24680815 | CASP8 |
| 10027 | 16.0833 | 0 | Unmethylated | 24680815 | CASP8 |
| 10028 | 16.0833 | 0 | Unmethylated | 24680815 | CASP8 |
| 10029 | 16.0833 | 0 | Unmethylated | 24680815 | CASP8 |
| 10030 | 16.0833 | 0 | Unmethylated | 24680815 | CASP8 |
| 10031 | 16.0833 | 0 | Unmethylated | 24680815 | CASP8 |
| 10032 | 16.0833 | 0 | Unmethylated | 24680815 | CASP8 |
| 10033 | 16.0833 | 0 | Unmethylated | 24680815 | CASP8 |
| 10034 | 16.0833 | 0 | Unmethylated | 24680815 | CASP8 |
| 10035 | 16.0833 | 0 | Unmethylated | 24680815 | CASP8 |
| 10036 | 16.0833 | 0 | Unmethylated | 24680815 | CASP8 |
| 10037 | 16.0833 | 0 | Unmethylated | 24680815 | CASP8 |
| 10038 | 16.0833 | 0 | Unmethylated | 24680815 | CASP8 |
| 10039 | 16.0833 | 0 | Unmethylated | 24680815 | CASP8 |

| | | | | | |
|---|---|---|---|---|---|
| 10040 | 16.0833 | 0 | Unmethylated | 24680815 | CASP8 |
| 10041 | 16.0833 | 0 | Unmethylated | 24680815 | CASP8 |
| 10042 | 16.0833 | 0 | Unmethylated | 24680815 | CASP8 |
| 10043 | 16.0833 | 0 | Unmethylated | 24680815 | CASP8 |
| 10044 | 16.0833 | 0 | Unmethylated | 24680815 | CASP8 |
| 10045 | 16.0833 | 0 | Unmethylated | 24680815 | CASP8 |
| 10046 | 16.0833 | 0 | Unmethylated | 24680815 | CASP8 |
| 10047 | 16.0833 | 0 | Unmethylated | 24680815 | CASP8 |
| 10048 | 16.0833 | 0 | Unmethylated | 24680815 | CASP8 |
| 10049 | 16.0833 | 0 | Unmethylated | 24680815 | CASP8 |
| 10050 | 16.0833 | 0 | Unmethylated | 24680815 | CASP8 |
| 10051 | 16.0833 | 0 | Unmethylated | 24680815 | CASP8 |
| 10052 | 16.0833 | 0 | Unmethylated | 24680815 | CASP8 |
| 10053 | 16.0833 | 0 | Unmethylated | 24680815 | CASP8 |
| 10054 | 16.0833 | 0 | Unmethylated | 24680815 | CASP8 |
| 10055 | 16.0833 | 0 | Unmethylated | 24680815 | CASP8 |
| 10056 | 16.0833 | 0 | Unmethylated | 24680815 | CASP8 |
| 10057 | 16.0833 | 0 | Unmethylated | 24680815 | CASP8 |
| 10058 | 16.0833 | 0 | Unmethylated | 24680815 | CASP8 |
| 10059 | 16.0833 | 0 | Unmethylated | 24680815 | CASP8 |
| 10060 | 16.0833 | 0 | Unmethylated | 24680815 | CASP8 |
| 10061 | 16.0833 | 0 | Unmethylated | 24680815 | CASP8 |
| 10062 | 16.0833 | 0 | Unmethylated | 24680815 | CASP8 |
| 10063 | 16.0833 | 0 | Unmethylated | 24680815 | CASP8 |
| 10064 | 16.0833 | 0 | Unmethylated | 24680815 | CASP8 |
| 10065 | 16.0833 | 0 | Unmethylated | 24680815 | CASP8 |
| 10066 | 16.0833 | 0 | Unmethylated | 24680815 | CASP8 |
| 10067 | 16.0833 | 0 | Unmethylated | 24680815 | CASP8 |
| 10068 | 16.0833 | 0 | Unmethylated | 24680815 | CASP8 |
| 10069 | 16.0833 | 0 | Unmethylated | 24680815 | CASP8 |
| 10070 | 16.0833 | 0 | Unmethylated | 24680815 | CASP8 |
| 10071 | 16.0833 | 0 | Unmethylated | 24680815 | CASP8 |
| 10072 | 16.0833 | 0 | Unmethylated | 24680815 | CASP8 |
| 10073 | 16.0833 | 0 | Unmethylated | 24680815 | CASP8 |
| 10074 | 16.0833 | 0 | Unmethylated | 24680815 | CASP8 |
| 10075 | 16.0833 | 0 | Unmethylated | 24680815 | CASP8 |
| 10076 | 16.0833 | 0 | Unmethylated | 24680815 | CASP8 |
| 10077 | 16.0833 | 0 | Unmethylated | 24680815 | CASP8 |
| 10078 | 16.0833 | 0 | Unmethylated | 24680815 | CASP8 |
| 10079 | 16.0833 | 0 | Unmethylated | 24680815 | CASP8 |
| 10080 | 16.0833 | 0 | Unmethylated | 24680815 | CASP8 |
| 10081 | 16.0833 | 0 | Unmethylated | 24680815 | CASP8 |
| 10082 | 16.0833 | 0 | Unmethylated | 24680815 | CASP8 |
| 10083 | 16.0833 | 0 | Unmethylated | 24680815 | CASP8 |
| 10084 | 16.0833 | 0 | Unmethylated | 24680815 | CASP8 |
| 10085 | 16.0833 | 0 | Unmethylated | 24680815 | CASP8 |
| 10086 | 16.0833 | 0 | Unmethylated | 24680815 | CASP8 |

| | | | | | |
|---|---|---|---|---|---|
| 10087 | 16.0833 | 0 | Unmethylated | 24680815 | CASP8 |
| 10088 | 16.0833 | 0 | Unmethylated | 24680815 | CASP8 |
| 10089 | 16.0833 | 0 | Unmethylated | 24680815 | CASP8 |
| 10090 | 16.0833 | 0 | Unmethylated | 24680815 | CASP8 |
| 10091 | 16.0833 | 0 | Unmethylated | 24680815 | CASP8 |
| 10092 | 16.0833 | 0 | Unmethylated | 24680815 | CASP8 |
| 10093 | 16.0833 | 0 | Unmethylated | 24680815 | CASP8 |
| 10094 | 16.0833 | 0 | Unmethylated | 24680815 | CASP8 |
| 10095 | 16.0833 | 0 | Unmethylated | 24680815 | CASP8 |
| 10096 | 16.0833 | 0 | Unmethylated | 24680815 | CASP8 |
| 10097 | 16.0833 | 0 | Unmethylated | 24680815 | CASP8 |
| 10098 | 16.0833 | 0 | Unmethylated | 24680815 | CASP8 |
| 10099 | 16.0833 | 0 | Unmethylated | 24680815 | CASP8 |
| 10100 | 0 | 1 | Unmethylated | 24680815 | DCR2 |
| 10101 | 0 | 1 | Unmethylated | 24680815 | DCR2 |
| 10102 | 0.2183 | 1 | Unmethylated | 24680815 | DCR2 |
| 10103 | 0.2183 | 1 | Unmethylated | 24680815 | DCR2 |
| 10104 | 0.5817 | 1 | Unmethylated | 24680815 | DCR2 |
| 10105 | 0.7508 | 1 | Unmethylated | 24680815 | DCR2 |
| 10106 | 0.7508 | 1 | Unmethylated | 24680815 | DCR2 |
| 10107 | 0.875 | 1 | Unmethylated | 24680815 | DCR2 |
| 10108 | 0.875 | 1 | Unmethylated | 24680815 | DCR2 |
| 10109 | 0.875 | 1 | Unmethylated | 24680815 | DCR2 |
| 10110 | 1.0417 | 1 | Unmethylated | 24680815 | DCR2 |
| 10111 | 1.0417 | 1 | Unmethylated | 24680815 | DCR2 |
| 10112 | 1.575 | 1 | Unmethylated | 24680815 | DCR2 |
| 10113 | 1.575 | 1 | Unmethylated | 24680815 | DCR2 |
| 10114 | 1.575 | 1 | Unmethylated | 24680815 | DCR2 |
| 10115 | 2.0333 | 1 | Unmethylated | 24680815 | DCR2 |
| 10116 | 2.0333 | 1 | Unmethylated | 24680815 | DCR2 |
| 10117 | 2.275 | 1 | Unmethylated | 24680815 | DCR2 |
| 10118 | 2.325 | 1 | Unmethylated | 24680815 | DCR2 |
| 10119 | 2.7167 | 1 | Unmethylated | 24680815 | DCR2 |
| 10120 | 4.8667 | 1 | Unmethylated | 24680815 | DCR2 |
| 10121 | 5.2083 | 1 | Unmethylated | 24680815 | DCR2 |
| 10122 | 5.8583 | 1 | Unmethylated | 24680815 | DCR2 |
| 10123 | 5.8583 | 1 | Unmethylated | 24680815 | DCR2 |
| 10124 | 5.8583 | 1 | Unmethylated | 24680815 | DCR2 |
| 10125 | 16.1667 | 0 | Unmethylated | 24680815 | DCR2 |
| 10126 | 16.1667 | 0 | Unmethylated | 24680815 | DCR2 |
| 10127 | 16.1667 | 0 | Unmethylated | 24680815 | DCR2 |
| 10128 | 16.1667 | 0 | Unmethylated | 24680815 | DCR2 |
| 10129 | 16.1667 | 0 | Unmethylated | 24680815 | DCR2 |
| 10130 | 16.1667 | 0 | Unmethylated | 24680815 | DCR2 |
| 10131 | 16.1667 | 0 | Unmethylated | 24680815 | DCR2 |
| 10132 | 16.1667 | 0 | Unmethylated | 24680815 | DCR2 |
| 10133 | 16.1667 | 0 | Unmethylated | 24680815 | DCR2 |

| | | | | | |
|---|---|---|---|---|---|
| 10134 | 16.1667 | 0 | Unmethylated | 24680815 | DCR2 |
| 10135 | 16.1667 | 0 | Unmethylated | 24680815 | DCR2 |
| 10136 | 16.1667 | 0 | Unmethylated | 24680815 | DCR2 |
| 10137 | 16.1667 | 0 | Unmethylated | 24680815 | DCR2 |
| 10138 | 16.1667 | 0 | Unmethylated | 24680815 | DCR2 |
| 10139 | 16.1667 | 0 | Unmethylated | 24680815 | DCR2 |
| 10140 | 16.1667 | 0 | Unmethylated | 24680815 | DCR2 |
| 10141 | 16.1667 | 0 | Unmethylated | 24680815 | DCR2 |
| 10142 | 16.1667 | 0 | Unmethylated | 24680815 | DCR2 |
| 10143 | 16.1667 | 0 | Unmethylated | 24680815 | DCR2 |
| 10144 | 16.1667 | 0 | Unmethylated | 24680815 | DCR2 |
| 10145 | 16.1667 | 0 | Unmethylated | 24680815 | DCR2 |
| 10146 | 16.1667 | 0 | Unmethylated | 24680815 | DCR2 |
| 10147 | 16.1667 | 0 | Unmethylated | 24680815 | DCR2 |
| 10148 | 16.1667 | 0 | Unmethylated | 24680815 | DCR2 |
| 10149 | 16.1667 | 0 | Unmethylated | 24680815 | DCR2 |
| 10150 | 16.1667 | 0 | Unmethylated | 24680815 | DCR2 |
| 10151 | 16.1667 | 0 | Unmethylated | 24680815 | DCR2 |
| 10152 | 16.1667 | 0 | Unmethylated | 24680815 | DCR2 |
| 10153 | 16.1667 | 0 | Unmethylated | 24680815 | DCR2 |
| 10154 | 16.1667 | 0 | Unmethylated | 24680815 | DCR2 |
| 10155 | 16.1667 | 0 | Unmethylated | 24680815 | DCR2 |
| 10156 | 16.1667 | 0 | Unmethylated | 24680815 | DCR2 |
| 10157 | 16.1667 | 0 | Unmethylated | 24680815 | DCR2 |
| 10158 | 16.1667 | 0 | Unmethylated | 24680815 | DCR2 |
| 10159 | 16.1667 | 0 | Unmethylated | 24680815 | DCR2 |
| 10160 | 16.1667 | 0 | Unmethylated | 24680815 | DCR2 |
| 10161 | 16.1667 | 0 | Unmethylated | 24680815 | DCR2 |
| 10162 | 16.1667 | 0 | Unmethylated | 24680815 | DCR2 |
| 10163 | 16.1667 | 0 | Unmethylated | 24680815 | DCR2 |
| 10164 | 16.1667 | 0 | Unmethylated | 24680815 | DCR2 |
| 10165 | 16.1667 | 0 | Unmethylated | 24680815 | DCR2 |
| 10166 | 16.1667 | 0 | Unmethylated | 24680815 | DCR2 |
| 10167 | 16.1667 | 0 | Unmethylated | 24680815 | DCR2 |
| 10168 | 16.1667 | 0 | Unmethylated | 24680815 | DCR2 |
| 10169 | 16.1667 | 0 | Unmethylated | 24680815 | DCR2 |
| 10170 | 16.1667 | 0 | Unmethylated | 24680815 | DCR2 |
| 10171 | 16.1667 | 0 | Unmethylated | 24680815 | DCR2 |
| 10172 | 16.1667 | 0 | Unmethylated | 24680815 | DCR2 |
| 10173 | 16.1667 | 0 | Unmethylated | 24680815 | DCR2 |
| 10174 | 16.1667 | 0 | Unmethylated | 24680815 | DCR2 |
| 10175 | 16.1667 | 0 | Unmethylated | 24680815 | DCR2 |
| 10176 | 16.1667 | 0 | Unmethylated | 24680815 | DCR2 |
| 10177 | 16.1667 | 0 | Unmethylated | 24680815 | DCR2 |
| 10178 | 16.1667 | 0 | Unmethylated | 24680815 | DCR2 |
| 10179 | 16.1667 | 0 | Unmethylated | 24680815 | DCR2 |
| 10180 | 16.1667 | 0 | Unmethylated | 24680815 | DCR2 |

| | | | | | |
|---|---|---|---|---|---|
| 10181 | 16.1667 | 0 | Unmethylated | 24680815 | DCR2 |
| 10182 | 16.1667 | 0 | Unmethylated | 24680815 | DCR2 |
| 10183 | 16.1667 | 0 | Unmethylated | 24680815 | DCR2 |
| 10184 | 16.1667 | 0 | Unmethylated | 24680815 | DCR2 |
| 10185 | 16.1667 | 0 | Unmethylated | 24680815 | DCR2 |
| 10186 | 16.1667 | 0 | Unmethylated | 24680815 | DCR2 |
| 10187 | 16.1667 | 0 | Unmethylated | 24680815 | DCR2 |
| 10188 | 16.1667 | 0 | Unmethylated | 24680815 | DCR2 |
| 10189 | 16.1667 | 0 | Unmethylated | 24680815 | DCR2 |
| 10190 | 16.1667 | 0 | Unmethylated | 24680815 | DCR2 |
| 10191 | 16.1667 | 0 | Unmethylated | 24680815 | DCR2 |
| 10192 | 16.1667 | 0 | Unmethylated | 24680815 | DCR2 |
| 10193 | 16.1667 | 0 | Unmethylated | 24680815 | DCR2 |
| 10194 | 16.1667 | 0 | Unmethylated | 24680815 | DCR2 |
| 10195 | 16.1667 | 0 | Unmethylated | 24680815 | DCR2 |
| 10196 | 16.1667 | 0 | Unmethylated | 24680815 | DCR2 |
| 10197 | 16.1667 | 0 | Unmethylated | 24680815 | DCR2 |
| 10198 | 16.1667 | 0 | Unmethylated | 24680815 | DCR2 |
| 10199 | 16.1667 | 0 | Unmethylated | 24680815 | DCR2 |
| 10200 | 16.1667 | 0 | Unmethylated | 24680815 | DCR2 |
| 10201 | 16.1667 | 0 | Unmethylated | 24680815 | DCR2 |
| 10202 | 16.1667 | 0 | Unmethylated | 24680815 | DCR2 |
| 10203 | 16.1667 | 0 | Unmethylated | 24680815 | DCR2 |
| 10204 | 16.1667 | 0 | Unmethylated | 24680815 | DCR2 |
| 10205 | 16.1667 | 0 | Unmethylated | 24680815 | DCR2 |
| 10206 | 16.1667 | 0 | Unmethylated | 24680815 | DCR2 |
| 10207 | 0.7025 | 1 | Methylated | 24680815 | DCR2 |
| 10208 | 0.7992 | 1 | Methylated | 24680815 | DCR2 |
| 10209 | 0.9 | 1 | Methylated | 24680815 | DCR2 |
| 10210 | 1.1833 | 1 | Methylated | 24680815 | DCR2 |
| 10211 | 1.8667 | 1 | Methylated | 24680815 | DCR2 |
| 10212 | 1.8667 | 1 | Methylated | 24680815 | DCR2 |
| 10213 | 2.9333 | 1 | Methylated | 24680815 | DCR2 |
| 10214 | 2.9333 | 1 | Methylated | 24680815 | DCR2 |
| 10215 | 3 | 1 | Methylated | 24680815 | DCR2 |
| 10216 | 3.075 | 1 | Methylated | 24680815 | DCR2 |
| 10217 | 3.075 | 1 | Methylated | 24680815 | DCR2 |
| 10218 | 3.5333 | 1 | Methylated | 24680815 | DCR2 |
| 10219 | 3.6333 | 1 | Methylated | 24680815 | DCR2 |
| 10220 | 4 | 1 | Methylated | 24680815 | DCR2 |
| 10221 | 4.3083 | 1 | Methylated | 24680815 | DCR2 |
| 10222 | 6.5167 | 1 | Methylated | 24680815 | DCR2 |
| 10223 | 6.5167 | 1 | Methylated | 24680815 | DCR2 |
| 10224 | 12 | 0 | Methylated | 24680815 | DCR2 |
| 10225 | 12 | 0 | Methylated | 24680815 | DCR2 |
| 10226 | 12 | 0 | Methylated | 24680815 | DCR2 |
| 10227 | 12 | 0 | Methylated | 24680815 | DCR2 |

| | | | | | |
|---|---|---|---|---|---|
| 10228 | 12 | 0 | Methylated | 24680815 | DCR2 |
| 10229 | 12 | 0 | Methylated | 24680815 | DCR2 |
| 10230 | 12 | 0 | Methylated | 24680815 | DCR2 |
| 10231 | 12 | 0 | Methylated | 24680815 | DCR2 |
| 10232 | 12 | 0 | Methylated | 24680815 | DCR2 |
| 10233 | 12 | 0 | Methylated | 24680815 | DCR2 |
| 10234 | 12 | 0 | Methylated | 24680815 | DCR2 |
| 10235 | 12 | 0 | Methylated | 24680815 | DCR2 |
| 10236 | 0.3392 | 1 | Methylated | 24680815 | CASP8 |
| 10237 | 0.85 | 1 | Methylated | 24680815 | CASP8 |
| 10238 | 0.9417 | 1 | Methylated | 24680815 | CASP8 |
| 10239 | 0.9417 | 1 | Methylated | 24680815 | CASP8 |
| 10240 | 1.0417 | 1 | Methylated | 24680815 | CASP8 |
| 10241 | 1.0417 | 1 | Methylated | 24680815 | CASP8 |
| 10242 | 1.2083 | 1 | Methylated | 24680815 | CASP8 |
| 10243 | 1.55 | 1 | Methylated | 24680815 | CASP8 |
| 10244 | 1.8167 | 1 | Methylated | 24680815 | CASP8 |
| 10245 | 1.8167 | 1 | Methylated | 24680815 | CASP8 |
| 10246 | 2.0833 | 1 | Methylated | 24680815 | CASP8 |
| 10247 | 2.1833 | 1 | Methylated | 24680815 | CASP8 |
| 10248 | 2.35 | 1 | Methylated | 24680815 | CASP8 |
| 10249 | 2.9333 | 1 | Methylated | 24680815 | CASP8 |
| 10250 | 2.9333 | 1 | Methylated | 24680815 | CASP8 |
| 10251 | 3.05 | 1 | Methylated | 24680815 | CASP8 |
| 10252 | 3.05 | 1 | Methylated | 24680815 | CASP8 |
| 10253 | 4.2417 | 1 | Methylated | 24680815 | CASP8 |
| 10254 | 5.9083 | 1 | Methylated | 24680815 | CASP8 |
| 10255 | 5.9083 | 1 | Methylated | 24680815 | CASP8 |
| 10256 | 11.9167 | 0 | Methylated | 24680815 | CASP8 |
| 10257 | 11.9167 | 0 | Methylated | 24680815 | CASP8 |
| 10258 | 11.9167 | 0 | Methylated | 24680815 | CASP8 |
| 10259 | 11.9167 | 0 | Methylated | 24680815 | CASP8 |
| 10260 | 11.9167 | 0 | Methylated | 24680815 | CASP8 |
| 10261 | 11.9167 | 0 | Methylated | 24680815 | CASP8 |
| 10262 | 11.9167 | 0 | Methylated | 24680815 | CASP8 |
| 10263 | 11.9167 | 0 | Methylated | 24680815 | CASP8 |
| 10264 | 11.9167 | 0 | Methylated | 24680815 | CASP8 |
| 10265 | 11.9167 | 0 | Methylated | 24680815 | CASP8 |
| 10266 | 11.9167 | 0 | Methylated | 24680815 | CASP8 |
| 10267 | 11.9167 | 0 | Methylated | 24680815 | CASP8 |
| 10268 | 11.9167 | 0 | Methylated | 24680815 | CASP8 |
| 10269 | 11.9167 | 0 | Methylated | 24680815 | CASP8 |
| 10270 | 11.9167 | 0 | Methylated | 24680815 | CASP8 |
| 10271 | 11.9167 | 0 | Methylated | 24680815 | CASP8 |
| 10272 | 0.9667 | 1 | Unmethylated | 24680815 | RASSF1 |
| 10273 | 0.9667 | 1 | Unmethylated | 24680815 | RASSF1 |
| 10274 | 1.5917 | 1 | Unmethylated | 24680815 | RASSF1 |

| | | | | | |
|---|---|---|---|---|---|
| 10275 | 1.7083 | 1 | Unmethylated | 24680815 | RASSF1 |
| 10276 | 6.5 | 1 | Unmethylated | 24680815 | RASSF1 |
| 10277 | 6.5 | 1 | Unmethylated | 24680815 | RASSF1 |
| 10278 | 6.5 | 1 | Unmethylated | 24680815 | RASSF1 |
| 10279 | 6.5 | 1 | Unmethylated | 24680815 | RASSF1 |
| 10280 | 12.9167 | 0 | Unmethylated | 24680815 | RASSF1 |
| 10281 | 12.9167 | 0 | Unmethylated | 24680815 | RASSF1 |
| 10282 | 12.9167 | 0 | Unmethylated | 24680815 | RASSF1 |
| 10283 | 12.9167 | 0 | Unmethylated | 24680815 | RASSF1 |
| 10284 | 12.9167 | 0 | Unmethylated | 24680815 | RASSF1 |
| 10285 | 12.9167 | 0 | Unmethylated | 24680815 | RASSF1 |
| 10286 | 12.9167 | 0 | Unmethylated | 24680815 | RASSF1 |
| 10287 | 12.9167 | 0 | Unmethylated | 24680815 | RASSF1 |
| 10288 | 12.9167 | 0 | Unmethylated | 24680815 | RASSF1 |
| 10289 | 12.9167 | 0 | Unmethylated | 24680815 | RASSF1 |
| 10290 | 12.9167 | 0 | Unmethylated | 24680815 | RASSF1 |
| 10291 | 12.9167 | 0 | Unmethylated | 24680815 | RASSF1 |
| 10292 | 12.9167 | 0 | Unmethylated | 24680815 | RASSF1 |
| 10293 | 12.9167 | 0 | Unmethylated | 24680815 | RASSF1 |
| 10294 | 12.9167 | 0 | Unmethylated | 24680815 | RASSF1 |
| 10295 | 12.9167 | 0 | Unmethylated | 24680815 | RASSF1 |
| 10296 | 12.9167 | 0 | Unmethylated | 24680815 | RASSF1 |
| 10297 | 12.9167 | 0 | Unmethylated | 24680815 | RASSF1 |
| 10298 | 12.9167 | 0 | Unmethylated | 24680815 | RASSF1 |
| 10299 | 12.9167 | 0 | Unmethylated | 24680815 | RASSF1 |
| 10300 | 12.9167 | 0 | Unmethylated | 24680815 | RASSF1 |
| 10301 | 12.9167 | 0 | Unmethylated | 24680815 | RASSF1 |
| 10302 | 12.9167 | 0 | Unmethylated | 24680815 | RASSF1 |
| 10303 | 12.9167 | 0 | Unmethylated | 24680815 | RASSF1 |
| 10304 | 12.9167 | 0 | Unmethylated | 24680815 | RASSF1 |
| 10305 | 12.9167 | 0 | Unmethylated | 24680815 | RASSF1 |
| 10306 | 12.9167 | 0 | Unmethylated | 24680815 | RASSF1 |
| 10307 | 12.9167 | 0 | Unmethylated | 24680815 | RASSF1 |
| 10308 | 12.9167 | 0 | Unmethylated | 24680815 | RASSF1 |
| 10309 | 12.9167 | 0 | Unmethylated | 24680815 | RASSF1 |
| 10310 | 12.9167 | 0 | Unmethylated | 24680815 | RASSF1 |
| 10311 | 12.9167 | 0 | Unmethylated | 24680815 | RASSF1 |
| 10312 | 12.9167 | 0 | Unmethylated | 24680815 | RASSF1 |
| 10313 | 12.9167 | 0 | Unmethylated | 24680815 | RASSF1 |
| 10314 | 12.9167 | 0 | Unmethylated | 24680815 | RASSF1 |
| 10315 | 12.9167 | 0 | Unmethylated | 24680815 | RASSF1 |
| 10316 | 12.9167 | 0 | Unmethylated | 24680815 | RASSF1 |
| 10317 | 12.9167 | 0 | Unmethylated | 24680815 | RASSF1 |
| 10318 | 12.9167 | 0 | Unmethylated | 24680815 | RASSF1 |
| 10319 | 12.9167 | 0 | Unmethylated | 24680815 | RASSF1 |
| 10320 | 12.9167 | 0 | Unmethylated | 24680815 | RASSF1 |
| 10321 | 12.9167 | 0 | Unmethylated | 24680815 | RASSF1 |

| | | | | | |
|---|---|---|---|---|---|
| 10322 | 12.9167 | 0 | Unmethylated | 24680815 | RASSF1 |
| 10323 | 12.9167 | 0 | Unmethylated | 24680815 | RASSF1 |
| 10324 | 0 | 1 | Methylated | 24680815 | RASSF1 |
| 10325 | 0 | 1 | Methylated | 24680815 | RASSF1 |
| 10326 | 0 | 1 | Methylated | 24680815 | RASSF1 |
| 10327 | 0.3133 | 1 | Methylated | 24680815 | RASSF1 |
| 10328 | 0.7467 | 1 | Methylated | 24680815 | RASSF1 |
| 10329 | 0.7467 | 1 | Methylated | 24680815 | RASSF1 |
| 10330 | 0.7467 | 1 | Methylated | 24680815 | RASSF1 |
| 10331 | 0.7467 | 1 | Methylated | 24680815 | RASSF1 |
| 10332 | 0.8917 | 1 | Methylated | 24680815 | RASSF1 |
| 10333 | 0.8917 | 1 | Methylated | 24680815 | RASSF1 |
| 10334 | 0.9833 | 1 | Methylated | 24680815 | RASSF1 |
| 10335 | 0.9833 | 1 | Methylated | 24680815 | RASSF1 |
| 10336 | 1.0583 | 1 | Methylated | 24680815 | RASSF1 |
| 10337 | 1.0583 | 1 | Methylated | 24680815 | RASSF1 |
| 10338 | 1.4917 | 1 | Methylated | 24680815 | RASSF1 |
| 10339 | 1.7833 | 1 | Methylated | 24680815 | RASSF1 |
| 10340 | 1.7833 | 1 | Methylated | 24680815 | RASSF1 |
| 10341 | 2.025 | 1 | Methylated | 24680815 | RASSF1 |
| 10342 | 2.025 | 1 | Methylated | 24680815 | RASSF1 |
| 10343 | 2.1667 | 1 | Methylated | 24680815 | RASSF1 |
| 10344 | 2.2667 | 1 | Methylated | 24680815 | RASSF1 |
| 10345 | 2.9417 | 1 | Methylated | 24680815 | RASSF1 |
| 10346 | 2.9417 | 1 | Methylated | 24680815 | RASSF1 |
| 10347 | 3.0583 | 1 | Methylated | 24680815 | RASSF1 |
| 10348 | 3.0583 | 1 | Methylated | 24680815 | RASSF1 |
| 10349 | 3.0583 | 1 | Methylated | 24680815 | RASSF1 |
| 10350 | 3.5167 | 1 | Methylated | 24680815 | RASSF1 |
| 10351 | 3.5167 | 1 | Methylated | 24680815 | RASSF1 |
| 10352 | 3.5167 | 1 | Methylated | 24680815 | RASSF1 |
| 10353 | 4.2417 | 1 | Methylated | 24680815 | RASSF1 |
| 10354 | 4.2417 | 1 | Methylated | 24680815 | RASSF1 |
| 10355 | 4.2417 | 1 | Methylated | 24680815 | RASSF1 |
| 10356 | 5.0083 | 1 | Methylated | 24680815 | RASSF1 |
| 10357 | 5.85 | 1 | Methylated | 24680815 | RASSF1 |
| 10358 | 5.85 | 1 | Methylated | 24680815 | RASSF1 |
| 10359 | 16 | 0 | Methylated | 24680815 | RASSF1 |
| 10360 | 16 | 0 | Methylated | 24680815 | RASSF1 |
| 10361 | 16 | 0 | Methylated | 24680815 | RASSF1 |
| 10362 | 16 | 0 | Methylated | 24680815 | RASSF1 |
| 10363 | 16 | 0 | Methylated | 24680815 | RASSF1 |
| 10364 | 16 | 0 | Methylated | 24680815 | RASSF1 |
| 10365 | 16 | 0 | Methylated | 24680815 | RASSF1 |
| 10366 | 16 | 0 | Methylated | 24680815 | RASSF1 |
| 10367 | 16 | 0 | Methylated | 24680815 | RASSF1 |
| 10368 | 16 | 0 | Methylated | 24680815 | RASSF1 |

| | | | | | |
|---|---|---|---|---|---|
| 10369 | 16 | 0 | Methylated | 24680815 | RASSF1 |
| 10370 | 16 | 0 | Methylated | 24680815 | RASSF1 |
| 10371 | 16 | 0 | Methylated | 24680815 | RASSF1 |
| 10372 | 16 | 0 | Methylated | 24680815 | RASSF1 |
| 10373 | 16 | 0 | Methylated | 24680815 | RASSF1 |
| 10374 | 16 | 0 | Methylated | 24680815 | RASSF1 |
| 10375 | 16 | 0 | Methylated | 24680815 | RASSF1 |
| 10376 | 16 | 0 | Methylated | 24680815 | RASSF1 |
| 10377 | 16 | 0 | Methylated | 24680815 | RASSF1 |
| 10378 | 16 | 0 | Methylated | 24680815 | RASSF1 |
| 10379 | 16 | 0 | Methylated | 24680815 | RASSF1 |
| 10380 | 16 | 0 | Methylated | 24680815 | RASSF1 |
| 10381 | 16 | 0 | Methylated | 24680815 | RASSF1 |
| 10382 | 16 | 0 | Methylated | 24680815 | RASSF1 |
| 10383 | 16 | 0 | Methylated | 24680815 | RASSF1 |
| 10384 | 16 | 0 | Methylated | 24680815 | RASSF1 |
| 10385 | 16 | 0 | Methylated | 24680815 | RASSF1 |
| 10386 | 16 | 0 | Methylated | 24680815 | RASSF1 |
| 10387 | 16 | 0 | Methylated | 24680815 | RASSF1 |
| 10388 | 16 | 0 | Methylated | 24680815 | RASSF1 |
| 10389 | 16 | 0 | Methylated | 24680815 | RASSF1 |
| 10390 | 16 | 0 | Methylated | 24680815 | RASSF1 |
| 10391 | 16 | 0 | Methylated | 24680815 | RASSF1 |
| 10392 | 16 | 0 | Methylated | 24680815 | RASSF1 |
| 10393 | 16 | 0 | Methylated | 24680815 | RASSF1 |
| 10394 | 16 | 0 | Methylated | 24680815 | RASSF1 |
| 10395 | 16 | 0 | Methylated | 24680815 | RASSF1 |
| 10396 | 16 | 0 | Methylated | 24680815 | RASSF1 |
| 10397 | 16 | 0 | Methylated | 24680815 | RASSF1 |
| 10398 | 16 | 0 | Methylated | 24680815 | RASSF1 |
| 10399 | 16 | 0 | Methylated | 24680815 | RASSF1 |
| 10400 | 16 | 0 | Methylated | 24680815 | RASSF1 |
| 10401 | 16 | 0 | Methylated | 24680815 | RASSF1 |
| 10402 | 16 | 0 | Methylated | 24680815 | RASSF1 |
| 10403 | 16 | 0 | Methylated | 24680815 | RASSF1 |
| 10404 | 16 | 0 | Methylated | 24680815 | RASSF1 |
| 10405 | 16 | 0 | Methylated | 24680815 | RASSF1 |
| 10406 | 16 | 0 | Methylated | 24680815 | RASSF1 |
| 10407 | 16 | 0 | Methylated | 24680815 | RASSF1 |
